# Cue identification in phenology: A case study of the predictive performance of current statistical tools

**DOI:** 10.1111/1365-2656.13038

**Published:** 2019-06-27

**Authors:** Emily G. Simmonds, Ella F. Cole, Ben C. Sheldon

**Affiliations:** ^1^ Department of Zoology Edward Grey Institute, University of Oxford Oxford UK; ^2^ Department of Mathematical Sciences and Centre for Biodiversity Dynamics Norwegian University of Science and Technology (NTNU) Trondheim Norway

**Keywords:** climate sensitivity profile, cue identification, GAM, great tits, growing degree days, penalized signal regression, phenology, sliding windows

## Abstract

1. Changes in the timing of life‐history events (phenology) are a widespread consequence of climate change. Predicting population resilience requires knowledge of how phenology is likely to change over time, which can be gained by identifying the specific environmental cues that drive phenological events. Cue identification is often achieved with statistical testing of candidate cues. As the number of methods used to generate predictions increases, assessing the predictive accuracy of different approaches has become necessary.
2. This study aims to (a) provide an empirical illustration of the predictive ability of five commonly applied statistical methods for cue identification (absolute and relative sliding time window analyses, penalized signal regression, climate sensitivity profiles and a growing degree‐day model) and (b) discuss approaches for implementing cue identification methods in different systems.
3. Using a dataset of mean clutch initiation timing in wild great tits (*Parus major*), we explored how the days of the year identified as most important, and the aggregate statistic identified as a cue, differed between statistical methods and with respect to the time span of data used. Each method's predictive capacity was tested using cross‐validation and assessed for robustness to varying sample size.
4. We show that the identified critical time window of cue sensitivity was consistent across four of the five methods. The accuracy and precision of predictions differed by method with penalized signal regression resulting in the most accurate and most precise predictions in our case. Accuracy was maximal for near‐future predictions and showed a relationship with time. The difference between predictions and observations systematically shifted across the study from preceding observations to lagging.
5. This temporal trend in prediction error suggests that the current statistical tools either fail to capture a key component of the cue–phenology relationship, or the relationship itself is changing through time in our system. These two influences need to be teased apart if we are to generate realistic predictions of phenological change. We recommend future phenological studies to challenge the idea of a static cue–phenology relationship and should cross‐validate results across multiple time periods.

## Graphical abstract

The authors give a case study of the predictive performance of five different phenological cue identification methods. They identify a temporal bias in prediction error, suggesting that the current statistical tools either fail to capture a key component of the cue–phenology relationship, or the relationship itself is changing through time. Photo credit: Emily G. Simmonds.

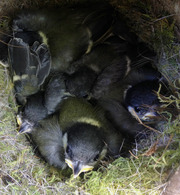

## 1 INTRODUCTION

Rapid climate change is causing shifts in the timing of annual peak resource availability for many animal populations across the world (Cook et al., 2012; Post, Pedersen, Wilmers, & Forchhammer, 2008; Singer & Parmesan, 2010; Visser, Holleman, & Gienapp, 2006; Visser, Marvelde, & Lof, 2012). Species can respond to these changes through evolution or by phenotypic plasticity in the timing of life‐history events. Phenotypic plasticity has often been indicated as the primary driver of interannual phenological change, for example in breeding and migration dates of birds, summarized in Charmantier and Gienapp (2014). Species are thought to either respond directly to environmental changes or to proxy cues which relate to optimal timing. When temperature sensitivity differs between species, interspecific interactions can be disrupted causing temporal mismatch between trophic levels. Such mismatches impact individual fitness, with poor matching reducing survival and reproductive success, potentially affecting population resilience (Lane, Kruuk, Charmantier, Murie, & Dobson, 2012; Plard et al., 2014; Reed, Jenouvrier, & Visser, 2013; Visser et al., 2006).

Predicting phenological change over time is an important element in assessing population resilience, since identifying potential trophic mismatches can highlight populations at risk. Prediction is becoming more common in ecological studies, particularly those including phenology (van Asch, Tienderen, Holleman, & Visser, 2007; Chuine & Beaubien, 2001; Cleland et al., 2007; Pau et al., 2011; Roy & Sparks, 2000). Before generating predictions, however, it is necessary to build an understanding of how species time their life‐history events. Life‐history events such as the onset of reproduction (Ardia, Cooper, & Dhondt, 2006; Charmantier et al., 2008), flowering (Cleland et al., 2007; Menzel et al., 2006), migration (Usui, Butchart, & Phillimore, 2017) and hibernation (Lane et al., 2012) have been linked to environmental variables (Parmesan, 2006). However, identifying the exact environmental cue or cues used is challenging. To establish causation between a proposed cue and phenology would require experimentation, preferably in the organism's natural environment. However, such experiments are logistically challenging, particularly for large and mobile species. As a result, for long‐term phenological studies, the primary tool for identifying environmental cues has been statistical analysis. There are two overarching approaches: phenomenological and mechanistic (Roberts, Tansey, Smithers, & Phillimore, 2015). Phenomenological approaches are based on statistical associations between observed data (Roberts et al., 2015). Mechanistic approaches are based on assuming known biological processes as drivers of variation. Both methods identify correlative rather than causal relationships. This impedes the teasing apart of real cues from confounding variables, particularly as many weather variables are highly autocorrelated both temporally and spatially. Of the different methods used, the phenomenological sliding time window and smoothing function‐based regressions, and the mechanistic growing degree‐day model (GDD), have been used most frequently.

Sliding time window analyses—a regression‐based approach (Hudson, 2010)—have been widely applied (Bailey & De Pol, 2016; Husby et al., 2010; Perrins & McCleery, 1989; Phillimore et al., 2013; van de Pol et al., 2016; van de Pol & Cockburn, 2011; Samplonius et al., 2018). This approach statistically identifies the critical time window in which an environmental variable explains the most variance in phenology. Typically, the explanatory variable is some measure of an abiotic weather variable across a temporal window; the response variable is the phenological variable of interest. The duration and temporal position of the window can be altered, and the explanatory power of each model compared. The temporal position is either tied to a reference calendar day (i.e. 20 May) or to the time event itself (i.e. days prior to event); these approaches are absolute sliding time window (SWA) analysis and relative sliding time window (SWR) analysis, respectively (van de Pol et al., 2016). SWAs vary in the annual lag between the window and the event, whereas SWRs are fixed relative to the phenological event, but calendar day can vary. The SWR has been proposed as an alternative to absolute methods to allow for individual or interannual variation in weather conditions experienced (van de Pol et al., 2016; van de Pol & Cockburn, 2011). Absolute methods (SWA; climate sensitivity profiles—CSP; and P‐spline signal regressions—PSR), which are tied to a calendar day, can identify good proxies for the actual cue, which can be useful for prediction, if the relationship between the cue and proxy is stable. However, they cannot identify a true biological cue. It is biologically implausible that the exact same period of calendar days, for example May temperature, influences the phenological event in all individuals or in all years. For example, in a particularly warm year the mean phenological event timing may fall prior to or within May. Consequently, the timing decision could not be influenced by this cue and therefore it cannot be a true biological cue, even if it were to correlate strongly with it (therefore being a good proxy). Relative windows provide some alternative to this in an attempt to access a more biologically meaningful cue by having temporally variable windows, for example temperature in the month prior to laying would influence lay date each year. Instead of being fixed to a calendar day, SWR methods are fixed to the phenological event, covering different calendar days each year (or for each individual) but having a fixed lag time between the cue and event.

Smoothing function methods are a more recent approach (Roberts, 2008; Roberts et al., 2015; Thackeray et al., 2016). They are also regression‐based analyses but employ smoothing functions and penalties to generate sensitivity profiles (Roberts, 2010). These analyses consider the influence of the environment on all days during a year rather than bounded windows. The PSR method (Roberts, 2008) and CSP method (Thackeray et al., 2016) are both examples of smoothing function approaches. Using these methods, it is possible to identify the most influential days of the year, based on the effect size.

Growing degree‐day (GDD) models were developed for plants (Chuine, 2000; Kramer, 1995; Rötzer, Grote, & Pretzsch, 2004), based on the assumption of linear relations between temperature and development due to enzyme activity (Bonhomme, 2000). Any temperatures exceeding a particular threshold are considered to contribute to development, and the influence of each degree over the threshold is accumulated until a second threshold is reached and the phenological event occurs. These models can be applied to animal systems although fewer direct developmental links to temperature might be expected for endothermic animals (Khaliq, Hof, Prinzinger, Böhning‐Gaese, & Pfenninger, 2014; McNab, 2012).

A fundamental implicit assumption across all of these methods is that the environmental cues driving phenology remain consistent across time. Many studies of phenology in long‐term systems continue to use the same cue identified previously to inform later analyses (Charmantier et al., 2008; Visser et al., 2006; Visser, Noordwijk, van Tinbergen, & Lessells, 1998). Alternatively, cues are defined using all data available or without consideration of the temporal distribution of the data (Husby et al., 2010; Perrins & McCleery, 1989; Phillimore et al., 2013; van de Pol et al., 2016; Thackeray et al., 2016). Both approaches assume that the cue, or the relationship between a proxy and timing, does not change over the time period considered. This assumption fails to account not only for the potential of changing cues over time but also for the influence of sample size on the cues identified.

Other methods exist in the cue identification toolkit, such as machine learning (Holloway, Kudenko, &Bell, 2018); however, the above are the most commonly applied and well developed. Previous studies have compared the performance of different methods in capturing environmental sensitivity (Phillimore et al., 2013; Roberts et al., 2015), finding largely congruent results across different methods. Similar cues were identified using GDD, SWA and PSR (Phillimore et al., 2013; Roberts et al., 2015). While predictive potential was inferred in these studies through *R* ^2^ values, the predictive capacity and accuracy of different methods were not compared. The aims of explanation and prediction are fundamentally different and models optimized for one will not necessarily perform well for the other. Therefore, it is timely to assess the predictive performance of cues identified for the aim of explanation as a predictive focus in ecology increases.

In this study, we address the following specific objectives, using a dataset of population mean annual laying date, collected over 55 years from a long‐term study of the great tit, as our phenology measure to:

1. Compare and contrast temperature cues identified (allowing variation in both the critical window and aggregate statistic of the cue) by five commonly used methods (sliding window absolute analysis (SWA), sliding window relative analysis (SWR), climate sensitivity profiles (CSP), penalized signal regression (PSR) and a growing degree‐day model (GDD)).
2. Assess how these identified cues vary depending on the length of the dataset and the precise time period used.
3. Use the temperature cues identified by the different methods and lengths of dataset to predict phenology for five‐year test datasets of observed data.
4. Evaluate the predictive performance of the different methods and interpret their biological meaning.

## 2 MATERIALS AND METHODS

### 2.1 The study system

The Wytham Woods great tit nest box population study has been conducted in a standardized way since 1960 (Perrins, 1979). Each breeding season (April–June), weekly nest box checks are carried out to provide data on nest stage, number of eggs and the onset of incubation. Clutch initiation date is determined by assuming a laying rate of one egg per day; therefore, it is possible to count backwards from the number of eggs counted on the weekly check to determine the date that the clutch was initiated. Species identity (four species of tit use the study nest boxes) is initially confirmed by weighing eggs, when at least three are present; great tit eggs can be confirmed by average egg weight of >1.3 g per egg. Clutch initiation timing, hereafter “lay date,” has been extensively studied in this population and strongly linked to spring temperature (Charmantier et al., 2008; Husby et al., 2010; van Noordwijk, McCleery, & Perrins, 1995; Perrins & McCleery, 1989). Therefore, we focus on testing temperature cues only in this study. We also exclude any lay dates more than 30 days after the first lay date of the year from analyses. This is to avoid inclusion of second or replacement clutches (Van Der Jeugd, Henk, & McCleery, 2002). All mean lay dates were rounded to the nearest whole day.

This study uses annual mean lay dates from 55 years (1961–2015); these were calculated from 14,372 individual nest observations. The mean number of nests per year was 256.6 (range 114–473). The mean standard deviation of lay dates in a given year was 7.8 days (range 4.4–13.4 days).

Our biological dataset has the following format (column names): year, mean lay date (in days since 1 April), mean lay date (dd/mm/yyyy), day of the month, month, day of the year.

### 2.2 Environmental data collection

Temperature data were collected by the Meteorological Office, as part of the National Climate Information Centre gridded daily data (grid point 447500E 202500N) (Hollis & McCarthy, 2017; Met Office, 2009). These data are available at a 5 km by 5 km resolution across the United Kingdom (UK). Daily maximum and minimum temperatures were recorded in weather stations across the UK and the mean of these taken to produce a daily mean temperature measure. Spatial and temporal interpolation for missing data was conducted by the Meteorological Office (Met Office, 2009). Environmental data were available from 1960 to 2015.

Our environmental dataset has the following format (column names) with daily resolution; date (dd/mm/yyyy), day of month, month, day of year, temperature (°C).

### 2.3 Statistical analyses

#### 2.3.1 Cue identification methods

The response variable used in all analyses was the mean annual lay date for the Wytham Woods great tit population.

#### 2.3.2 Sliding time window analyses

The premise of sliding time window analyses is that there is an optimum time period in which the focal species is most strongly influenced by abiotic conditions. To identify this time period, environmental conditions, such as precipitation or temperature, are aggregated across a period of days (the time “window”). The duration and calendar position of the window are then altered to generate a series of candidate windows and associated environmental conditions, which are the explanatory variable. The number of candidates chosen can be exhaustive, testing all possible durations and positions within some bounds (van de Pol et al., 2016) or restricted to only a few a priori chosen candidates (Husby et al., 2010; Perrins, 1965). A regression‐based analysis (typically a linear model) is then used to determine the explanatory power of each candidate window. Model selection, with a focus on explanatory performance, is then conducted to determine the preferred model from the candidates. The aggregate statistic used in this method is typically a sum, mean (e.g. of the daily mean, minimum, or maximum temperature), minimum (e.g. the minimum mean daily temperature reached in a focal window) or maximum (e.g. the maximum mean daily temperature reached in a focal window) environmental value, or the slope of environmental change across the window (the gradient of a linear model of daily mean temperature against date within the focal window).

#### 2.3.3 Absolute sliding time window (SWA)

In SWA, the position of the candidate window occurs at the same point in the calendar year every year, for example always from the 10 April to 20 May. In other words, it holds an absolute position not influenced by phenological timing.

In this study, we chose to implement the SWA as an exhaustive analysis. For this analysis, we used a reference day of the 20 May to bound exploration to windows that occur prior to that date, meaning all windows are characterized in “days prior to 20 May.” Windows were allowed to vary in length from a single day up to 365 days. The whole year was used for consistency with other methods in this study (CSP and PSR) and to reduce a priori restrictions. It also allows influence of autumn or winter temperatures to be detected if these are important, as has been previously reported for some species (e.g. Thackeray et al., 2016). Four aggregate temperature statistics were tested (mean, minimum and maximum temperatures, and the slope of temperature change across each window—calculated as the gradient from a linear model of temperature against day in window). All aggregate statistics were calculated from data of mean daily temperature. For instance, the minimum and maximum are the most extreme mean daily temperatures that occurred during a particular window. Model selection was performed using the AICc (Akaike information criterion (Akaike, 1973) with a small sample size correction); the preferred model was identified by having the lowest AICc compared to the baseline model, an intercept‐only linear model of annual mean lay dates. The SWA analysis was run using the R package “climwin” (Bailey & De Pol, 2016; van de Pol et al., 2016). This package was designed to standardize the process of fitting sliding time windows to phenological data. It allows for exhaustive exploratory analyses across a variety of aggregate statistics.

#### 2.3.4 Relative sliding time window (SWR)

SWR is similar in principle to SWA; however, the candidate windows are not tied to a calendar day, but to the phenological event itself. All windows are defined in “days prior to the phenological event” and occur on different calendar days each year.

In the same way as for SWA analyses, the time windows were allowed to vary in length from one to 365 days, and we tested aggregate temperature statistics of the mean, minimum and maximum temperature, and slope of temperature change across each window. Model selection was again conducted using AICc (with small sample size correction) compared to the baseline model, an intercept‐only linear model of annual lay date means. SWR analyses were also run using the “climwin” package in R.

#### 2.3.5 Climate sensitivity profile (CSP)

The CSP technique was first introduced by Thackeray et al. (2016), and we follow their general methodology here. This method takes a measure of an environmental variable on a single day, for example daily mean or maximum temperature, and regresses this against the phenological event of interest in turn for every day of a year. The first day taken is the day prior to a reference day. Here, we used 20 May as our reference. We began with mean temperature for the 20 May and regressed this against the annual mean lay dates (using the lm function in R), iterating backwards through time up to 365 days prior to 20 May. For each regression, we saved the coefficient value (the slope of the relationship between temperature and phenology) and the *R* ^2^. These coefficient and *R* ^2^ values were each passed through a GAM using the R package “mgcv” (Wood, 2001; Wood & Wood, 2015) to smooth the values across time. These smoothed functions are used to identify the calendar days of greatest influence on phenology. This period is defined as a period of consecutive days on which the coefficient and the *R* ^2^ values exceed the lower and upper quantiles, defined as greater or equal to the lowest 2.5% and highest 97.5% of values following the method used in Thackeray et al. (2016).

To run this method, our environmental data were reformatted so that each row was a year and each column was a day prior to 20 May, with entries being daily mean temperature. While Thackeray et al. propose using the date on which 95% of individuals have initiated laying (DOY95) as a response, we use annual mean lay date for consistency with the other methods trialled here. We also test using DOY95 to compare using the method as intended by the authors (see Figure S4).

#### 2.3.6 P‐spline signal regression (PSR)

P‐spline signal regression for phenological cue identification was introduced by Roberts (2008). This method works on a similar principle to the CSP methodology, but instead of a two‐step process there is a single smoothing and coefficient creating step. This method regresses all 365 days of temperature against the response, simultaneously creating partial coefficients (slope of the relationship between temperature and phenology). These partial coefficients are smoothed by penalizing for differences in consecutive days. The inclusion of many explanatory variables in a single analysis is addressed through a data reduction phase in order to reduce the high dimensionality. This is achieved by creating a B‐spline basis, creating a series of piecemeal polynomials (curves) joined at knots. These knots must be specified and cannot exceed one less than the sample size. The combination of B‐splines with a difference penalty results in P‐splines (penalized B‐splines), which penalizes differences between B‐splines to prevent overfitting. The level of difference penalty is chosen through cross‐validation. Here, we take the order of the B‐spline basis and the difference penalty from that described in Roberts (2008) and Roberts et al. (2015), cubic and first order, respectively. The approach can be implemented directly using a GAM. We ran the PSR using the “mgcv” R package. The GAM is run on the raw phenology data and climate data indexed to the reference date of 20 May. Our climate data were arranged in the same way as for the CSP method. The whole year of temperature can be used to subsequently predict phenology in given years. The most important days of temperature influence on phenology in the year may be identified by as those with partial coefficients greater than or less than zero by more than two times their standard error.

#### 2.3.7 Growing degree day (GDD)

The GDD model we implement here is a three‐parameter thermal‐time model (also used in Phillimore et al., 2013). The parameters used in this model are: start date, minimum threshold temperature and the cumulative GDD requirement. The environmental values begin being accumulated from the start date onwards; here, we use mean daily temperatures. Every degree above the minimum threshold temperature is cumulatively summed until the cumulative GDD requirement is reached, at which point the phenological event is predicted to occur. These parameters were optimized to minimize the sum of squared differences between the predicted annual mean lay date and the observed mean lay dates. The sum of squares was calculated using a linear model with the observed phenology as an explanatory variable and predicted phenology as the response. Optimization was performed using a generalized simulated annealing optimizer through the “GenSA” R package (Xiang, Gubian, Suomela, & Hoeng, 2013). A wide area of parameter space is searched with bounds for each parameter of start dates from 1 to 200 (year day on which temperature starts being counted); minimum temperature from 1°C to 10°C; and cumulative GDD requirement of 50°C to 1,000°C. Parameters were bootstrapped 1,000 times using the “boot” package in R (Canty & Ripley, 2017; Davidson & Hinkley, 1997), and percentile bootstrap confidence intervals for each parameter were produced to capture uncertainty in the parameter estimation. It should be noted that the aggregate statistic used for GDD models is cumulative sum of temperature in contrast to predominantly mean temperature in other methods.

#### 2.3.8 Identification of critical time windows and aggregate statistics

In this study, we identified cues using several different data subsets to address the question of whether the time period covered by the data alters the cue identified. To answer this question, the data were divided into three subsets: a 50‐year training dataset (1961–2010), the first 25 years of data (1961–1985) and the last 25 years of data (1986–2010). Data from 2011 to 2015 were retained separately to use as a test dataset for predictive analyses. An optimal temperature cue was identified using each of the five methods detailed above, by running each method following its own protocol.

To assess the amount of variance explained by the identified cues, we ran linear models with annual mean lay date as a response variable and the cues identified as the explanatory variables. For the SWA, SWR and CSP methods, the cue identified is a temperature variable. For the GDD and PSR methods, the cue identified is the date on which the cumulative GDD requirement is reached or the fitted date from the PSR model. For each of the linear models, the adjusted *R* ^2^ value was calculated as an indication of the amount of variance in the annual mean lay date that has been explained by the focal cue.

#### 2.3.9 Prediction of lay dates using identified cues

Only four of the five methods were used predictively in this study because the SWR cue is defined based on the timing of the phenological event, and therefore, to identify the cue, the event must have already occurred. As a result, prediction using this method was not possible. For the SWA and CSP methods, predictions of annual mean lay date timing were generated from linear models of the identified cues against phenology, based on the observed temperature values from the test years. For PSR, predictions were generated from the P‐spline GAM using all days of temperature in the 365 days preceding 20 May. For the GDD model, the temperature data from the test years were passed through the GDD equation using previously optimized parameter values to identify the date in each year on which the cumulative GDD requirement was reached. Prediction intervals were also generated for all methods using the results of the linear models or GAM for SWA, CSP and PSR. However, for the GDD method prediction intervals were generated by predicting using the lower and upper bounds of estimates of each parameter from bootstrapped confidence intervals.

We explored the influence of the time period of the training data relative to the test data and the length of the training data on predictive performance at several resolutions. In order to tease apart these different influences, three sets of predictions were generated to answer specific questions:

1. How does the number of years of data influence predictive performance? To address this, we subdivided the 50‐year training dataset into eight smaller training datasets, creating a total of nine datasets. The eight smaller datasets decrease in 10‐year increments in both directions (i.e. 1971–2010 and 1961–2000), down to two 10‐year datasets of the earliest and latest decade. Cues were identified for each training dataset using the four methods where prediction is possible (SWA, CSP, PSR and GDD). Predictions of mean annual lay date were then generated for test datasets, which cover the 5 years directly following the end of each training dataset (i.e. for a training dataset of years 1961–2000 the test years are 2001–2005).
2. How does the temporal distance between the dataset and predicted years interact with data length to influence predictive performance? To address this question, we used the same data subsets from above but instead of using the 5 years following the end of the training dataset as test years we used only 2011–2015. For datasets ending in 2010 (five of the nine), this was the same analysis as above. This allowed the influence of years of study to be distinguished from the influence of temporal lag between data and predictions.
3. How accurate and precise are predictions from the SWA, CSP, PSR and GDD methods across our dataset? To address this, we conducted K‐fold (in this instance 5‐fold) cross‐validation to determine predictive performance when accounting for stochasticity in the test years of data. We split the original 55‐year dataset (1961–2015) into five‐year subsets and in turn predicted one five‐year subset from the parameters generated by the remaining 50 years (e.g. 1961–1965 could be held as a test dataset and 1966–2015 data used to predict phenology in the test years).

To quantify the accuracy of the phenological predictions and compare across different methods and data subsets, the mean absolute error (MAE) was calculated for each set of five predicted years (2011–2015). The MAE is the mean of the positive value of all discrepancies between the predicted lay dates and the observed lay dates (error) across the five test years. The larger the MAE, the greater the discrepancy between predicted and observed phenology. In addition to the MAE, we also calculated the raw discrepancy (retaining sign of error) between the predicted and observed phenology during the K‐fold cross‐validation (the mean prediction error). This allowed an exploration of bias in the predictions, which would not be captured by MAE.

Precision of predictions was represented by prediction intervals. The widths of prediction intervals were compared, with wider intervals indicating lower precision. We also explored how well the precision is captured by calculating the proportion of times the observed value fell within the prediction interval for each set of five test years (coverage of the prediction interval).

## 3 RESULTS

### 3.1 Critical time windows of sensitivity

**Figure 1:**
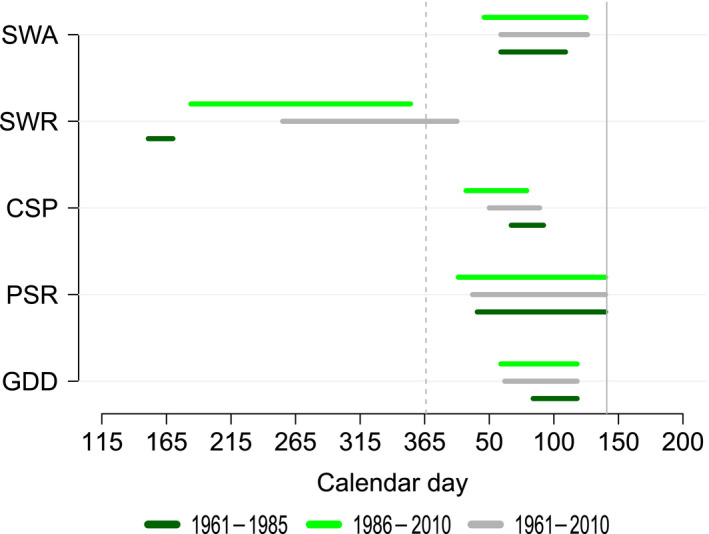
Temporal critical windows for great tit egg‐laying phenology identified by different statistical methods and timeframes of data. Vertical dotted line shows 1 January, and solid vertical line indicates 20 May, the reference day for absolute methods; absolute sliding window analysis (SWA), climate sensitivity profile (CSP) and penalized signal regression (PSR). Growing degree‐day model (GDD) is plotted relative to the mean lay date across years and the relative sliding window analysis (SWR) relative to the reference date of 20 May

We found considerable variability in the exact days identified as the “critical window” of environmental sensitivity; the window length and position varied based on the method used and the time period covered by the dataset (Figure 1). The timing of the critical window was broadly similar between the SWA, PSR, CSP and GDD methods, for all time periods. However, the SWR method identified windows considerably earlier than all other methods, more than 200 days earlier at the most extreme. The SWR method showed large differences in window timing depending on the dataset used, with older data producing earlier windows. Time windows identified using the whole long‐term training dataset (1961–2010) were typically midway between the early data and late data, with the exception of SWR where the window was different from both early and late. In addition, for all methods except SWR, the windows for the latter half of our data (1986–2010) began earlier and were longer in duration than the windows identified using older data.

Relationships between the identified cue and the mean annual lay date varied from explaining 41% (CSP) to 77% (PSR) of the variance in the response variable. Three of the five (SWA, SWR and PSR) explained approx. 70%, or greater, of the variation (Table 1). When selecting for a model which maximizes explanatory power, dissimilar environmental cues can still produce similar results and consequently be difficult to choose between. This is not surprising in highly explorative analyses such as this; consequently, *R* ^2^ should be used with caution.

**Table 1:** Summary statistics for linear models of identified cues and lay date for all methods

Method	Time period	Aggregate statistic	Intercept	Slope	*SE*	*R* ^2^	Window open	Window close
SWA	Whole dataset	Mean across window	163.91	−6.06	0.54	0.72	81	14
SWR	Whole dataset	Mean across window	167.33	−5.24	0.49	0.69	250	165
CSP	Whole dataset	Mean across window	135.78	−3.06	0.52	0.41	90	51
PSR	Whole dataset	Daily mean	181.08	NA	NA	0.77	102.95	0.00

								°C min	°C total
GDD	Whole dataset	Sum	0.09	0.91	10.06	0.66	61.53	1.03	355.50

Note

Shows time period of data used, intercept of the regression, slope of the relationship, standard error (*SE*), adjusted *R* ^2^ (*R* ^2^), window open and window close (in days prior to reference day of 20 May). The minimum threshold temperature and cumulative GDD requirement are also presented.

### 3.2 How accurate is predicted phenology?

**Figure 2:**
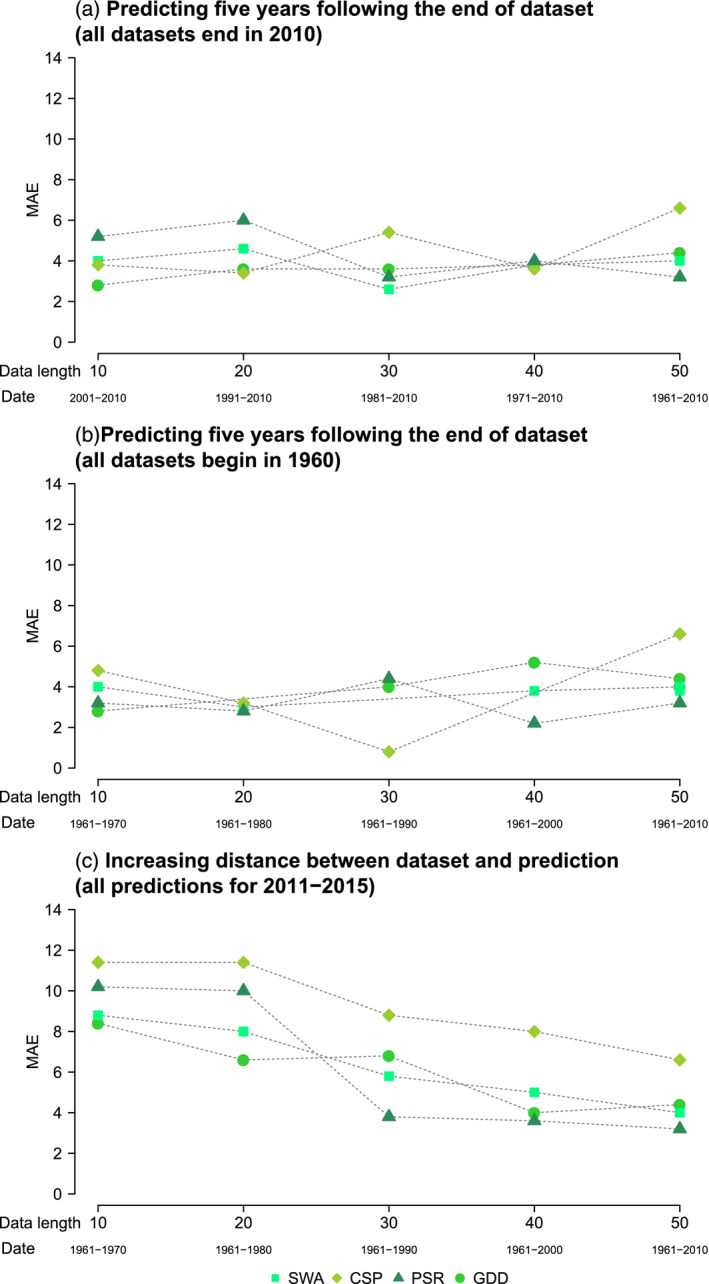
Mean absolute error (MAE) of model predictions using different amounts of data. Error between predictions and observations shown for different training dataset lengths from 10 to 50 years, in 10‐year increments beginning. (a) Beginning at 2001–2010 and extending backwards in time. (b and c) Beginning at 1961–1970 and extending forwards in time. Predicted years are the 5 years directly following the end of training data (a and b) or 2011–2015 (c)

Error between predicted and observed phenology was lowest for near‐future predictions—those where test years occur directly after the end of the training dataset (Figure 2a,b). These predictions had MAE that did not exceed 7 days. The standard deviation in mean annual lay dates across the study period was 7.2 days; therefore, all near‐future predictions had error which was lower than the standard deviation of the data as a whole.

Sample size did not appear to have a strong influence on predictive accuracy. Mean absolute error remained consistent across five different sample sizes from 10 to 50 years in Figure 2(a,b). There was a slight reduction of MAE with increasing sample size in Figure 2c. However, this also coincided with decreasing temporal lag between data and predictions. The larger the temporal lag between data and predictions, the greater the error up to 12 days MAE. When data were collected relative to when it is predicting had a greater impact on accuracy than the total number of years of data. The PSR method had the lowest mean MAE and consequently the highest accuracy of all methods in K‐fold cross‐validation (Table 2). SWA, GDD and CSP also had mean MAE of <7 days.

**Table 2:** Summary of predictive accuracy and precision of K‐fold cross‐validation

Method	MAE	PI width	% observations in PI (coverage)
SWA	3.35	15.29	96
CSP	4.60	21.03	93
PSR	3.16	6.73	58
GDD	6.60	66.13	82

**Figure 3:**
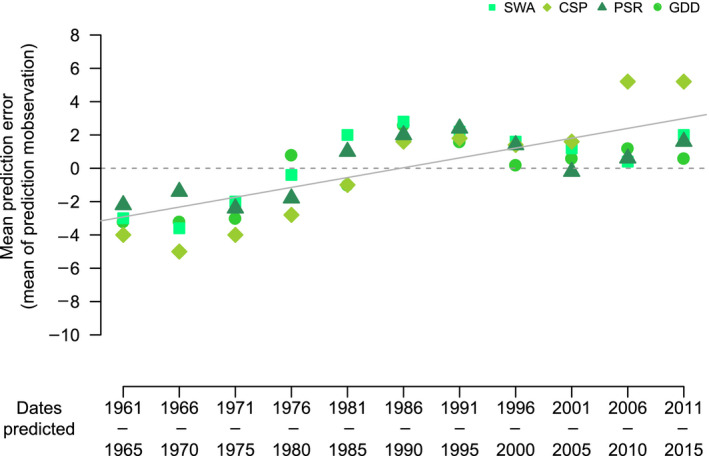
Plot of the error from K‐fold cross‐validation (mean difference between predicted and observed mean annual lay date). Error is plotted against the time period of the five‐year test dataset it is generated for. Plotted solid line is a fitted least‐squares line, dashed line indicates 0

While MAE was consistent across all sets of test years for near‐future predictions, the mean prediction error (mean of the raw values of prediction minus observed) indicated systematic bias in accuracy (Figure 3). Non‐random structure was present in the error. Predictions for the earliest half of the study period tended to be earlier than observations, and predictions for the latter half of the study period tended to be later than observations. This structure was described by using a fitted least‐squares line with study year as an explanatory variable: 50% of the variation in the error was explained by year of study (quantified by multiple *R* ^2^). While the GDD method did demonstrate bias in the prediction error, in the same direction as all other methods, the error for this method did not appear to follow a linear trend. If only the regression‐based analyses were included, the amount of variation explained by the linear term increased to 66%. The fitted line in Figure 3 crossed 0 at approximately the mid‐point of our study (1986).

### 3.3 How precise are our predictions of phenology?

**Figure 4:**
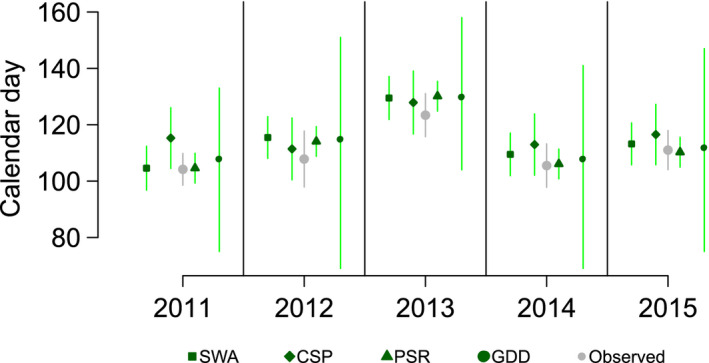
Predicted and observed mean annual lay dates. Predictions generated from different methods, using the whole long‐term dataset, are plotted against the observed lay dates. Vertical lines represent 95% prediction intervals or the standard deviation for observed data

The amount of uncertainty (how precise the predictions are) in predicted phenology—the width of prediction intervals—varied substantially between methods (Table 2 and Figure 4) from 6.73 days for the PSR method to 66.13 for the GDD method. There was no clear association between width of prediction intervals and either number of years of data or distance from the data and predictions (Table 2). The range of the 95% prediction intervals of all methods except PSR was more than double the within (7.8 days)‐ and between (7.2 days)‐year standard deviation in lay dates for this population.

Within methods, there was no association between how accurate and how precise predictions were, that is sets of test years with higher accuracy in predictions did not consistently have tighter prediction intervals. In contrast, between methods those with higher accuracy (PSR and SWA) were also more precise (have tighter prediction intervals—Table 2). The PSR method was both the most accurate and the most precise method, based on prediction interval width. However, the width of a prediction interval alone does not give us a complete picture of how precise a method is. It is also important to know whether the estimated prediction interval does accurately capture the uncertainty in a particular method. To quantify this, we calculated the proportion of prediction intervals which contained the observed value (coverage). For 95% prediction intervals, we would expect on average 95% of the intervals to overlap the observed value. From Table 2, it is clear that this was not always the case when predicting outside of the original dataset (the training dataset). The proportion of prediction intervals that contained the observed value in this study ranged from 100% to 0% (Table S2), but was most interesting for the K‐fold cross‐validation because these results take account of stochasticity in individual sets of test years by having 11 sets of test years (55 predicted years in total). For the K‐fold cross‐validation, the SWA and CSP methods had approximately 95% of observations falling within the prediction intervals, as would be expected. The GDD method had 82% of observations within the prediction intervals, whereas the PSR method had a particularly low proportion of observations within prediction intervals, 58%. This is much lower than expected even with stochastic variation. Despite the seemingly high precision and absolute accuracy of this method, it appears that precision in the predictions was not being correctly quantified.

## 4 DISCUSSION

### 4.1 Identified temperature cues differ between methods and over time but explanatory power of cues remains high

We have shown, using multiple analyses on the same dataset, that the identified temporal windows of environmental sensitivity vary in their position and duration dependent on the statistical method and the time period of data used (Figure 1). While there was some temporal overlap between the windows identified by SWA, CSP, PSR and GDD, the SWR method identified critical windows of temperature sensitivity that were 50–250 days earlier than other methods. The amount of temporal overlap in cues identified by different methods was highly influenced by the time period of data used to identify the cue. If only the most recent 25 years were used, the results of the SWA, CSP, PSR and GDD methods were largely congruent, as found in previous comparisons of statistical methods for cue identification (Phillimore et al., 2013; Roberts et al., 2015). However, when the earliest 25 years of data were used, more marked between‐method differences emerged. This appears to be driven by different susceptibility of the methods to the input data, with SWR having the greatest variation in cues identified. It should be noted that when CSP is employed using DOY95, as suggested in Thackeray et al. (2016), the windows identified by this method are more variable (see Figure S4). For all methods, excluding SWR, the windows identified by the most recent half of the data all began earlier in the year and had a longer duration than those identified by the earlier half. This is as would be expected as the birds are shifting their lay dates earlier in the year.

Despite the difference in cues identified by different methods, explanatory power of the different cues remained fairly high (excluding CSP). Using metrics based on the ability to explain variances such as AIC or *R* ^2^ may not be sufficient to distinguish between correlated cues. In the worst case, the *R* ^2^ values can be overly optimistic in highly explorative studies and should be interpreted with caution. As a result, we recommend, for prediction in particular, to assess how well the identified cues perform for prediction to determine whether the identified cues hold for future years or novel conditions.

Temporal movement of the cue identified across our study period data could indicate a shift in the actual temperature cue over time (Figure 1). This could operate through evolution of the cue–phenology relationship. The consistent (expect for SWR) shift to an earlier beginning of identified critical windows for more recent data could give support to evolution to use a different cue. However, an alternative possibility is that the statistical methods could be identifying proxies for the actual cue used by great tits and that under climatic change the relationship between these proxies and the actual cue are shifting, leading to a change in the proxy identified as the best predictor. It may also be the case that none of the statistical methods we use identify either the precise cue or a consistent proxy and that the cue identified is simply the best predictor of variance in lay dates for that particular time period and that this is altered depending on the exact years included.

We found (Figure 1) that the cue identified by SWR method was notably different to all other techniques, which identified very similar windows here and in previous comparison studies (Hudson, 2010; Phillimore et al., 2013; Roberts et al., 2015). This indicates that the relative approach can also be problematic in phenological cue identification.

The SWR approach has been used successfully to understand climate predictors of egg size in fairy wrens (Langmore, Bailey, Heinsohn, Russell, & Kilner, 2016). A variant on a relative approach was also successfully used to look at the timing of incubation onset relative to clutch completion (Simmonds, Sheldon, Coulson, & Cole, 2017); here, the temperature windows were tied to clutch completion rather than a calendar date but the lag between the cue and incubation onset remained variable. However, relative approaches should be used with caution. Due to the linear regression basis of these methods the strongest effect size, *R* ^2^, and lowest AIC values will be produced when there is variability in the explanatory variable and this variability correlates with the response variable, that is high values of temperature correspond with either early or late lay dates and low temperatures with the opposite. When using relative windows, it is likely that the time period preceding the phenological event will have similar temperature values for all years. For instance, if laying commences soon after particular temperatures are reached, regardless of their exact yearly timing. In this case, the explanatory power of windows identified close to the lay date will be low, because there will be lower variability in the explanatory variable than in the response variable. Only when a difference between temperatures for early and late years occurs will the explanatory power increase. This is likely to occur at periods of seasonal transition, for example, the onset of spring or winter. At this point, years with early lay dates will have their relative window cross these transitions prior to later years, generating strong temperature differences, a linear relationship and potentially temperatures which can explain variance in lay date. However, what has been identified is unlikely to be a cue for laying; instead, it is a statistical artefact of the method being used. The location of the SWR windows identified here, around the autumn and winter onsets, suggests that the statistical artefact discussed above might be the cause of the erroneous cues identified in this study.

Being aware of the statistical limitations of any methods used is vital for conducting analyses based on these methods. If used in a threshold rather than regression‐based format, relative windows might provide a suitable alternative to absolute methods, which can never identify the precise cues being used. Equally, a failure to identify a precise cue, if the proxy is good, may not be an impairment to many analyses. But relative windows used in a regression format to identify phenological cues will not statistically identify a true cue and cannot be used predictively, so risk being misleading in this context.

### 4.2 Predictive accuracy is highest for near‐future predictions

We have shown that the accuracy of predictions of phenological timing are influenced more by the temporal distance between the data used to identify the cue and the years being predicted, than by the number of years in the sample. When predicting the five years following the end of the data used to identify the cue, the prediction error remained consistent despite reductions in sample size (Figure 2a,b). This trend was consistent across all methods trialled. A reduction in MAE with increasing sample size was only seen in tandem with a reduction in the temporal distance between dataset end and predicted years (Figure 2c).

It is not surprising that near‐future predictions have higher accuracy than those further from the data used to parameterize the models. Extrapolating statistical relationships beyond the data used to estimate them can always be problematic as the identified relationships may not hold under novel conditions. Our results suggest that this may be the case in the Wytham Woods great tit population. When cues and cue–phenology relationships identified by data early in our study years are used to predict years later in the study, predictive accuracy is reduced. Such a pattern indicates that either the cue itself, or the cue–phenology relationship, is changing over the period of our study; further work should address the consequences of such changes.

The suggestion that biological systems, in particular phenological relationships, are not static is further supported by Figure 3. Our results show temporal autocorrelation in prediction error from K‐fold cross‐validation. Predictions earlier in our study years tended to be earlier than observed phenology; in contrast, predictions later in our study years tended to be later than observed phenology, with a single linear term (including prediction error from regression‐based methods only) explaining up to 66% of the variation observed. The mechanistic GDD method also showed the same bias in error but with a nonlinear trend. Therefore, a directional temporal component of the system has not been captured by our current analyses. One possibility is evolution of the cue–phenology relationship over the period of our study (c. 30 great tit generations). Previous work on another great tit population suggests that evolution of reaction norms may be hampered by low heritability and the sex‐limited nature of laying date (Ramakers, Gienapp, & Visser, 2018). However, exploration of evolution to use different cues (e.g. shifting to be sensitive to cues earlier in the year) has not yet been explored. Another possibility is that our models have not identified the true cue used by great tits to time phenology. If the true cue and the identified cue respond differently to climate change, then predictions will diverge over time. As a result, the impact of current climate change on phenology would not be correctly captured in our models and could lead to a temporal bias. A potential cause of this could be that all methods discussed here and the majority of studies (Charmantier et al., 2008; Husby et al., 2010; Perrins & McCleery, 1989; Visser et al., 1998) focus solely on the role of abiotic cues. It seems highly unlikely that biotic cues play no role in the phenology of other species. If in reality a biotic cue drives great tit phenology, this could create a pattern such as the one shown here. Teasing apart these two potential causes requires quantification of selection on the cue–phenology relationship, or validating cue identification experimentally (e.g. Lambrechts, Perret, Maistre, Perret, Maistre, & Blondel, 1999; Schaper et al., 2012; Schaper, Rueda, Sharp, Dawson, & Visser, 2011).

### 4.3 How precise predictions are differs by method and shows a between‐method association with accuracy

The predictive precision of methods varied from over 100 days (GDD method) to fewer than 7 days (PSR method). The very wide prediction intervals of the GDD method (Figure 4 and Table S2), which were several times larger than those of the other methods, likely stem from the need to estimate uncertainty of three parameters for this method (start date, minimum threshold temperature and the cumulative GDD requirement). This is in contrast to two (intercept and slope) for the regression‐based approaches. The PSR method was shown to be both the most precise and most accurate method. However, the precision in this method was not correctly quantified. This was demonstrated by the prediction intervals including the observed values only 58% of the time (Table 2). 58% is a much lower coverage than would be expected from a 95% prediction interval, even when allowing for the fact it will rarely cover exactly 95% (we would not expect coverage of <80% (Altman & Krzywinski, 2018)). This overestimation of precision in PSR could in part be influenced by the temporal trend in error as the error in PSR predictions for the middle years of our study was close to 0 (Figure 3); if the temporal trend in PSR is corrected, estimation of how precise the method is could also improve. This highlights the importance of cross‐validation and quantifying the accuracy and precision of predictions. Both the SWA and CSP methods had errors of approximately one week (across year standard deviation was 7 days). They also had prediction intervals of roughly two (SWA) or three (CSP) weeks. These intervals seem to represent the precision well in these methods, when considered across the whole study period (Table 2). These methods could provide rather uninformative predictions about phenology in this population, given that the precision of predictions is greater than twice the standard deviation of the data. A prediction interval of 15 days would imply that 95% of the observed mean lay dates were equally as plausible as the predicted estimate. As a result, little insight would be gained into the change in phenology over time.

When generating predictions, it is essential to consider which is most important for a particular question (accuracy or precision). Achieving a balance between these different predictive measures is important. One step to achieving this, possibly the most important, is to quantify these metrics and to be aware of the limitations of the method being used to predict. The results of this study demonstrate that the accuracy and precision of predictions relating to seasonal phenology are influenced by the method used and the distance between predictions and the data used to generate them. For near‐future predictions, all of the methods trialled here produced predictions with good accuracy but variable precision. If precision of predictions is not fully considered, this could create misleading predictions and misleading conclusions about the future of populations. However, with the right consideration of how the underlying statistical methods operate and cross‐validation of predictions, usable predictive outputs can be generated. In the case of our study system, accounting for the additional temporal trend that is not included in our models will also be essential to creating useable predictions.

All of the specific results here are generated from one study system and may not hold for other species or systems. However, the potential for variation in results generated by the different methods, shown here, is something that should be considered in all phenological analyses. This study is a step along a continual process of assessment and critique of the state of the art in ecological methods. As methodological approaches, computing power and data availability improve, we should remain aware of the limitations of the statistical tools we use, especially when applied to purposes beyond their original design. Here, we have provided an empirical illustration of how variable common cue identification methods can be in a predictive context. We hope that our recommendations below can act as best practice in the light of the fact that all populations will likely produce different results.

To create a theoretical understanding of the performance of these methods, simulation studies could be appealing. However, there are some difficulties with using small‐scale simulation studies when looking at phenological cue identification. The main problem is that we do not yet know the underlying cue that drives phenology. As a result, we have to assume a cue and a relationship between that cue and phenology in order to simulate data. As each cue identification method assumes a different underlying cue (e.g. temperature in a fixed window, temperature sum up to a threshold or temperature during the entire year), the cue chosen in order to simulate the data will inevitably have a strong influence on which method performs best. Extensive simulation studies would be a welcome next step to further investigating the performance of statistical cue identification tools. In the meantime, we recommend several key steps to follow when implementing cue identification methods on empirical data:

- Previously identified cues from populations should be reassessed when new data become available. By doing this, the assumptions that cues are static across time and that the cues identified previously remain reliable can be avoided (Charmantier et al., 2008; Visser et al., 2006).
- The merits of different statistical cue identification methods should be considered when applying them. Our results suggest that—for this dataset—the PSR and SWA methods were the most accurate and precise, with SWA having the more accurately estimated precision.
- More robust evaluation techniques need to be implemented as standard. In particular, K‐fold cross‐validation of predictions from the method chosen should be conducted and accuracy and precision should be quantified and reported. This can be easily implemented in some R packages such as climwin (Bailey & De Pol, 2016; van de Pol et al., 2016).
- More flexible approaches allowing windows that vary in length and timing across years should be explored. These could act as more realistic alternatives to absolute and relative regression‐based approaches, which both have theoretical flaws. In order to improve our confidence in cue identification (including exploring biotic cues), research effort is required to teasing apart the causes of the temporal autocorrelation in prediction error.

Cue identification models are increasingly being used predictively (Morin et al., 2009; van de Pol et al., 2016; Roberts et al., 2015; Thackeray et al., 2016), and consequently, it is timely to assess of the accuracy of such predictions. Our results suggest some fundamental issues with our current toolkit, particularly the inadequacy of the relative sliding window method for identification of phenological cues. They also demonstrate, through the temporal trend in predictive error, that our current tools either miss a key component of the cue–phenology relationship or the relationship is changing through time for some systems. Future phenological studies should challenge the idea of a static cue–phenology relationship and should cross‐validate results across multiple time periods.

## AUTHORS' CONTRIBUTIONS

E.G.S. conceived the ideas, designed the methodology, conducted the analyses and led the writing of this manuscript. E.G.S., E.F.C. and B.C.S. have all contributed to data collection. E.F.C. and B.C.S. provided detailed feedback on the methodological approaches and results throughout the analysis. All authors contributed critically to the drafts and gave final approval for publication.

## Data Availability

The biological and environmental data, and the R code used for this study are available as at the following GitHub repository: (https://github.com/emilygsimmonds/Cue_Identification) and archived in Zenodo: https://doi.org/10.5281/zenodo.2838825 (Simmonds, 2019). The environmental data were made available under open government licence by the MET Office https://www.metoffice.gov.uk/climate/uk/data/ukcp09/datasets.

## References

[jane13038-bib-0001] Akaike, H. (1973). Information theory and en extension of the maximum likelihood principle. 2nd International symposium on information theory (pp. 267–281).

[jane13038-bib-0002] Altman, N. , & Krzywinski, M. (2018). Predicting with confidence and tolerance. Nature Methods, 15(11), 843–845. 10.1038/s41592-018-0196-7 30377373

[jane13038-bib-0003] Ardia, D. R. , Cooper, C. B. , & Dhondt, A. A. (2006). Warm temperatures lead to early onset of incubation, shorter incubation periods and greater hatching asynchrony in tree swallows at the extremes of their range. Journal of Avian Biology, 37, 137–142.

[jane13038-bib-0004] Bailey, L. D. , & Van De Pol, M. (2016). Climwin: An R toolbox for climate window analysis. PLoS ONE, 11(12), e0167980. 10.1371/journal.pone.0167980 27973534 PMC5156382

[jane13038-bib-0005] Bonhomme, R. (2000). Bases and limits to using ‘degree.Day’ units. European Journal of Agronomy, 13(1), 1–10. 10.1016/S1161-0301(00)00058-7

[jane13038-bib-0006] Canty, A. , & Ripley, B. (2017). Boot: Bootstrap R (S‐Plus) functions.

[jane13038-bib-0007] Charmantier, A. , & Gienapp, P. (2014). Climate change and timing of avian breeding and migration: Evolutionary versus plastic changes. Evolutionary Applications, 7(1), 15–28. 10.1111/eva.12126 24454545 PMC3894895

[jane13038-bib-0008] Charmantier, A. , McCleery, R. H. , Cole, L. R. , Perrins, C. , Kruuk, L. E. , & Sheldon, B. C. (2008). Adaptive phenotypic plasticity in response to climate change in a wild bird population. Science, 320(5877), 800–803. 10.1126/science.1157174 18467590

[jane13038-bib-0009] Chuine, I. (2000). A unified model for budburst of trees. Journal of Theoretical Biology, 207(3), 337–347. 10.1006/jtbi.2000.2178 11082304

[jane13038-bib-0010] Chuine, I. , & Beaubien, E. G. (2001). Phenology is a major determinant of tree species range. Ecology Letters, 4(5), 500–510. 10.1046/j.1461-0248.2001.00261.x

[jane13038-bib-0011] Cleland , E. E. , Chuine , I. , Menzel , A. , … M. D. (2007). Shifting plant phenology in response to global change. Trends in Ecology & Evolution, 22(7), 357–365. 10.1016/j.tree.2007.04.003. 17478009

[jane13038-bib-0012] Cook, B. I. , Wolkovich, E. M. , Davies, T. J. , Ault, T. R. , Betancourt, J. L. , Allen, J. M. , … Travers, S. E. (2012). Sensitivity of spring phenology to warming across temporal and spatial climate gradients in two independent databases. Ecosystems, 15(8), 1283–1294. 10.1007/s10021-012-9584-5

[jane13038-bib-0013] Davidson, A. C. , & Hinkley, D. V. (1997). Bootstrap methods and their applications. Cambridge, UK: Cambridge University Press.

[jane13038-bib-0014] Hollis, D. , & McCarthy, M. (2017). Met office gridded and regional land surface climate observation datasets. Centre for Environmental Data Analysis. Retrieved from https://catalogue.ceda.ac.uk/uuid/87f43af9d02e42f483351d79b3d6162a

[jane13038-bib-0015] Holloway, P. , Kudenko, D. , & Bell, J. R. (2018). Dynamic selection of environmental variables to improve the prediction of aphid phenology: A machine learning approach. Ecological Indicators, 88, 512–521. 10.1016/j.ecolind.2017.10.032.

[jane13038-bib-0016] Hudson, I. L. (2010). Interdisciplinary approaches: Towards new statistical methods for phenological studies. Climatic Change, 100(1), 143–171. 10.1007/s10584-010-9859-9

[jane13038-bib-0017] Husby, A. , Nussey, D. H. , Visser, M. E. , Wilson, A. J. , Sheldon, B. C. , & Kruuk, L. E. B. (2010). Contrasting patterns of phenotypic plasticity in reproductive traits in two great tit (*Parus major*) populations. Evolution, 64(8), 2221–2237. 10.1111/j.1558-5646.2010.00991.x 20298465

[jane13038-bib-0018] Khaliq, I. , Hof, C. , Prinzinger, R. , Böhning‐Gaese, K. , & Pfenninger, M. (2014). Global variation in thermal tolerances and vulnerability of endotherms to climate change. Proceedings of the Royal Society B: Biological Sciences, 281(1789), 20141097–20141097. 10.1098/rspb.2014.1097 PMC4100521 25009066

[jane13038-bib-0019] Kramer, K. (1995). Phenotypic plasticity of the phenology of seven European tree species in relation to climatic warming. Plant, Cell & Environment, 18(2), 93–104. 10.1111/j.1365-3040.1995.tb00356.x

[jane13038-bib-0020] Lambrechts, M. M. , Perret, P. , Maistre, M. , & Blondel, J. (1999). Do experiments with captive non‐domesticated animals make sense without population field studies? A case study with blue tits' breeding time. Proceedings of the Royal Society B: Biological Sciences, 266(1426), 1311–1315.

[jane13038-bib-0021] Lane, J. E. , Kruuk, L. E. B. , Charmantier, A. , Murie, J. O. , & Dobson, F. S. (2012). Delayed phenology and reduced fitness associated with climate change in a wild hibernator. Nature, 489(7417), 554–557. 10.1038/nature11335 22878721

[jane13038-bib-0022] Langmore, N. E. , Bailey, L. D. , Heinsohn, R. G. , Russell, A. F. , & Kilner, R. M. (2016). Egg size investment in superb fairy‐wrens: Helper effects are modulated by climate. Proceedings of the Royal Society B: Biological Sciences, 283(1843), 1–8. 10.1098/rspb.2016.1875 PMC5136590 27903872

[jane13038-bib-0023] McNab, B. K. (2012). Extreme measures: The ecological energetics of birds and mammals. Chicago, IL: The University of Chicago Press.

[jane13038-bib-0024] Menzel, A. , Sparks, T. H. , Estrella, N. , Koch, E. , Aasa, A. , Ahas, R., … Zust, A. (2006). European phenological response to climate change matches the warming pattern. Global Change Biology, 12(10), 1969–1976. 10.1111/j.1365-2486.2006.01193.x

[jane13038-bib-0025] Met Office (2009). UKCP09 gridded observation datasets. Retrieved from https://www.metoffice.gov.uk/climate/uk/data/ukcp09

[jane13038-bib-0026] Morin, X. , Lechowicz, M. J. , Augspurger, C. , O'keefe, J. , Viner, D. , & Chuine, I. (2009). Leaf phenology in 22 North American tree species during the 21st century. Global Change Biology, 15(4), 961–975. 10.1111/j.1365-2486.2008.01735.x

[jane13038-bib-0027] Parmesan, C. (2006). Ecological and evolutionary responses to recent climate change. Annual Review of Ecology, Evolution and Systematics, 37(1), 637–669. 10.1146/annurev.ecolsys.37.091305.110100

[jane13038-bib-0028] Pau, S. , Wolkovich, E. M. , Cook, B. I. , Davies, T. J. , Kraft, N. J. B. , Bolmgren, K. , … Cleland, E. E. (2011). Predicting phenology by integrating ecology, evolution and climate science. Global Change Biology, 17(12), 3633–3643. 10.1111/j.1365-2486.2011.02515.x

[jane13038-bib-0029] Perrins, C. M. (1965). Fluctuations and clutch‐size in the great tit, *Parus major* L. Journal of Animal Ecology, 34(3), 601–647.

[jane13038-bib-0030] Perrins, C. M. (1979). British tits. London, UK: Collins.

[jane13038-bib-0031] Perrins, C. M. , & McCleery, R. H. (1989). Laying dates and clutch size in the great tit. The Wilson Journal of Ornithology, 101(2), 236–253.

[jane13038-bib-0032] Phillimore, A. B. , Proios, K. , O'Mahony, N. , Bernard, R. , Lord, A. M. , Atkinson, S. , & Smithers, R. J. (2013). Inferring local processes from macro‐scale phenological pattern: A comparison of two methods. Journal of Ecology, 101(3), 774–783. 10.1111/1365-2745.12067

[jane13038-bib-0033] Plard, F. , Gaillard, J. M. , Coulson, T. , Hewison, A. J. , Delorme, D. , Warnant, C. , & Bonenfant, C. (2014). Mismatch between birth dates and vegetation phenology slows the demography of roe deer. PLoS Biology, 12(4), 1–8. 10.1371/journal.pbio.1001828 PMC3972086 24690936

[jane13038-bib-0034] Post, E. , Pedersen, C. , Wilmers, C. C. , & Forchhammer, M. C. (2008). Warming, plant phenology and the spatial dimension of trophic mismatch for large herbivores. Proceedings of the Royal Society B: Biological Sciences, 275(1646), 2005–2013. 10.1098/rspb.2008.0463 PMC2596357 18495618

[jane13038-bib-0035] Ramakers, J. J. C. , Gienapp, P. , & Visser, M. E. (2018). Phenological mismatch drives selection on elevation, but not on slope, of breeding time plasticity in a wild songbird. Evolution, 73(2), 175–187. 10.1111/evo.13660 30556587 PMC6519030

[jane13038-bib-0036] Reed, T. , Jenouvrier, S. , & Visser, M. E. (2013). Phenological mismatch strongly affects individual fitness but not population demography in a woodland passerine. Journal of Animal Ecology, 82(1), 131–144. 10.1111/j.1365-2656.2012.02020.x 22862682

[jane13038-bib-0037] Roberts, A. M. I. (2008). Exploring relationships between phenological and weather data using smoothing. International Journal of Biometeorology, 52(6), 463–470. 10.1007/s00484-007-0141-4 18193297

[jane13038-bib-0038] Roberts, A. M. I. (2010). Smoothing methods. In M. R. Keatley , & I. L. Hudson (Eds.), Phenological research: Methods for environmental and climate change analysis (pp. 255–270). Netherlands: Springer.

[jane13038-bib-0039] Roberts, A. M. I. , Tansey, C. , Smithers, R. J. , & Phillimore, A. B. (2015). Predicting a change in the order of spring phenology in temperate forests. Global Change Biology, 21(7), 2603–2611. 10.1111/gcb.12896 25731862 PMC4964954

[jane13038-bib-0040] Rötzer, T. , Grote, R. , & Pretzsch, H. (2004). The timing of bud burst and its effect on tree growth. International Journal of Biometeorology, 48(3), 109–118. 10.1007/s00484-003-0191-1 14564495

[jane13038-bib-0041] Roy, D. B. , & Sparks, T. H. (2000). Phenology of British butterflies and climate change. Global Change Biology, 6(4), 407–416. 10.1046/j.1365-2486.2000.00322.x

[jane13038-bib-0042] Samplonius, J. M. , Bartošová, L. , Burgess, M. D. , Bushuev, A. V. , Eeva, T. , Ivankina, E. V. , … Both, C. (2018). Phenological sensitivity to climate change is higher in resident than in migrant bird populations among European cavity breeders. Global Change Biology, 24(8), 3780–3790. 10.1111/gcb.14160 29691942

[jane13038-bib-0043] Schaper, S. V. , Dawson, A. , Sharp, P. J. , Gienapp, P. , Caro, S. P. , & Visser, M. E. (2012). Increasing temperature, not mean temperature, is a cue for avian timing of reproduction. The American Naturalist, 179(2), E55–E69. 10.1086/663675 22218320

[jane13038-bib-0044] Schaper, S. V. , Rueda, C. , Sharp, P. J. , Dawson, A. , & Visser, M. E. (2011). Spring phenology does not affect timing of reproduction in the great tit (*Parus major*). The Journal of Experimental Biology, 214, 3664–3671. 10.1242/jeb.059543 21993796

[jane13038-bib-0045] Simmonds, E. (2019). emilygsimmonds/Cue_Identification: Cue identification in phenology code (Version Version1). Zenodo, 10.5281/zenodo.2838825

[jane13038-bib-0046] Simmonds, E. G. , Sheldon, B. C. , Coulson, T. , & Cole, E. F. (2017). Incubation behaviour adjustments, driven by ambient temperature variation, improve synchrony between hatch dates and caterpillar peak in a wild bird population. Ecology and Evolution, 7, 9415–9425. 29187978 10.1002/ece3.3446 PMC5696398

[jane13038-bib-0047] Singer, M. C. , & Parmesan, C. (2010). Phenological asynchrony between herbivorous insects and their hosts: Signal of climate change or pre‐existing adaptive strategy? Philosophical Transactions of the Royal Society B: Biological Sciences, 365(1555), 3161–3176. 10.1098/rstb.2010.0144 PMC2981947 20819810

[jane13038-bib-0048] Thackeray, S. J. , Henrys, P. A. , Hemming, D. , Bell, J. R. , Botham, M. S. , Burthe, S. , … Wanless, S. (2016). Phenological sensitivity to climate across taxa and trophic levels. Nature, 535(7611), 241–245. 10.1038/nature18608 27362222

[jane13038-bib-0049] Usui, T. , Butchart, S. H. M. , & Phillimore, A. B. (2017). Temporal shifts and temperature sensitivity of avian spring migratory phenology: A phylogenetic meta‐analysis. Journal of Animal Ecology, 86(2), 250–261. 10.1111/1365-2656.12612 PMC6849580 27859281

[jane13038-bib-0050] van Asch, M. , van Tienderen, P. H. , Holleman, L. J. M. , & Visser, M. E. (2007). Predicting adaptation of phenology in response to climate change, an insect herbivore example. Global Change Biology, 13(8), 1596–1604. 10.1111/j.1365-2486.2007.01400.x

[jane13038-bib-0051] van de Pol, M. , Bailey, L. D. , McLean, N. , Rijsdijk, L. , Lawson, C. R. , & Brouwer, L. (2016). Identifying the best climatic predictors in ecology and evolution. Methods in Ecology and Evolution, 7(10), 1246–1257. 10.1111/2041-210X.12590

[jane13038-bib-0052] van de Pol, M. , & Cockburn, A. (2011). Identifying the critical climatic time window that affects trait expression. The American Naturalist, 177(5), 698–707. 10.1086/659101 21508615

[jane13038-bib-0053] Van Der Jeugd, V. , Henk, P. , & McCleery, R. H. (2002). Effects of spatial autocorrelation, natal philopatry and phenotypic plasticity on the heritability of laying date. Journal of Evolutionary Biology, 15(3), 380–387. 10.1046/j.1420-9101.2002.00411.x

[jane13038-bib-0054] van Noordwijk, A. J. , McCleery, R. H. , & Perrins, C. M. (1995). Selection for the timing of great tit breeding in relation to caterpillar growth and temperature. Journal of Animal Ecology, 64(4), 451–458. 10.2307/5648

[jane13038-bib-0055] Visser, M. E. , Holleman, L. J. M. , & Gienapp, P. (2006). Shifts in caterpillar biomass phenology due to climate change and its impact on the breeding biology of an insectivorous bird. Oecologia, 147(1), 164–172. 10.1007/s00442-005-0299-6 16328547

[jane13038-bib-0056] Visser, M. E. , Noordwijk, A. J. , van Tinbergen, J. M. , & Lessells, C. M. (1998). Warmer springs lead to mistimed reproduction in great tits (*Parus major*). Proceedings of the Royal Society of London. Series B: Biological Sciences, 265(1408), 1867–1870. 10.1098/rspb.1998.0514

[jane13038-bib-0057] Visser, M. E. , te Marvelde, L. , & Lof, M. E. (2012). Adaptive phenological mismatches of birds and their food in a warming world. Journal of Ornithology, 153(S1), 75–84. 10.1007/s10336-011-0770-6

[jane13038-bib-0058] Wood, S. N. (2001). Mgcv: GAMs and Generalized Ridge Regression for R. R News.

[jane13038-bib-0059] Wood, S. N. , & Wood, M. S. (2015). Package ‘Mgcv’. R package version: 1–7. Retrieved from http://cran.stat.auckland.ac.nz/web/packages/mgcv/mgcv.pdf%0A https://cran.r‐project.org/web/packages/mgcv/mgcv.pdf

[jane13038-bib-0060] Xiang, Y. , Gubian, S. , Suomela, B. , & Hoeng, J. (2013). Generalized simulated annealing for global optimization: The GenSA package. R Journal, 5(1), 13–28. 10.32614/RJ-2013-002

