# Supplementary material for: Cue identification in phenology: A case study of the predictive performance of current statistical tools
Source: J Anim Ecol. 2019 Jun 27;88(9):1428–40. doi: 10.1111/1365-2656.13038 (PMC8629117; doi:10.1111/1365-2656.13038)
Supplement: Supplementary file 1 [file JANE-88-1428-s001.docx]

### Supporting information for

***Cue identification in phenology: a case study of the predictive performance of current statistical tools***

**Authors:** Emily G. Simmonds^1,2*^, Ella F. Cole^1^, and Ben C. Sheldon^1^

^1.^ Edward Grey Institute, Department of Zoology, University of Oxford, UK OX1 3PS

^2.^ Department of Mathematical Sciences and Centre for Biodiversity Dynamics, Norwegian University of Science and Technology (NTNU), Norway

^*^Corresponding author: email address [emilygsimmonds@gmail.com](mailto:emilygsimmonds@gmail.com)

Address: Department of Mathematical Sciences, Norwegian University of Science and Technology (NTNU), Norway

**This file contains:**

| **S1** Full climate profiles for CSP | 2 |
| --- | --- |
| **S2** Full GAM smooth results PSR | 10 |
| **S3** Plot of critical time windows (DOY95 for CSP) | 12 |
| **S4** Full version of manuscript Table 1 | 13 |
| **S5** Summary of predictive precision from all data subsets | 15 |

#### S1 – Full climate profiles for CSP

##### Mean annual lay date as response variable

Below we present the results of the GAMs performed on the slope coefficient and R^2^ values for each data subset when mean annual lay date is the response variable.


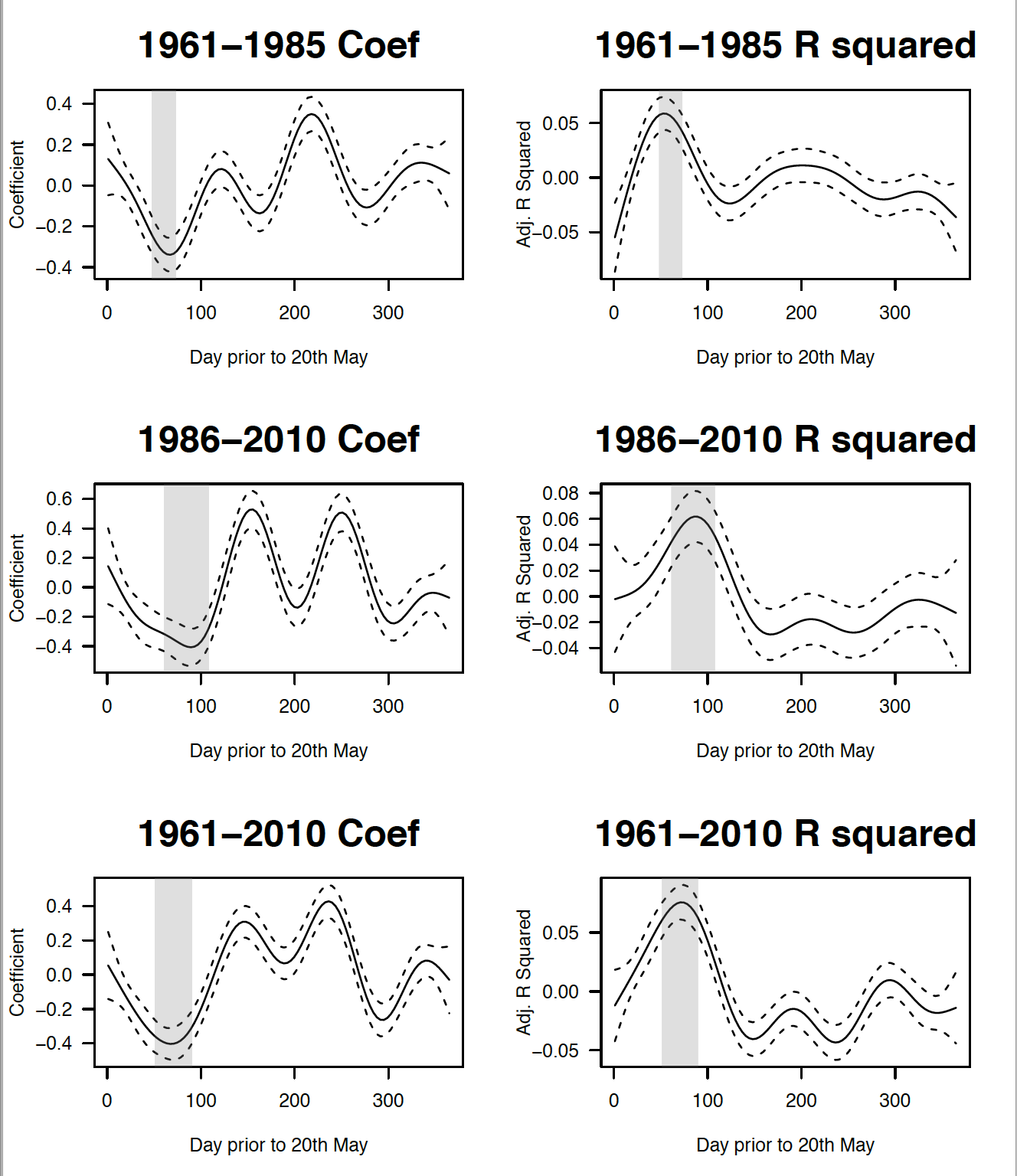


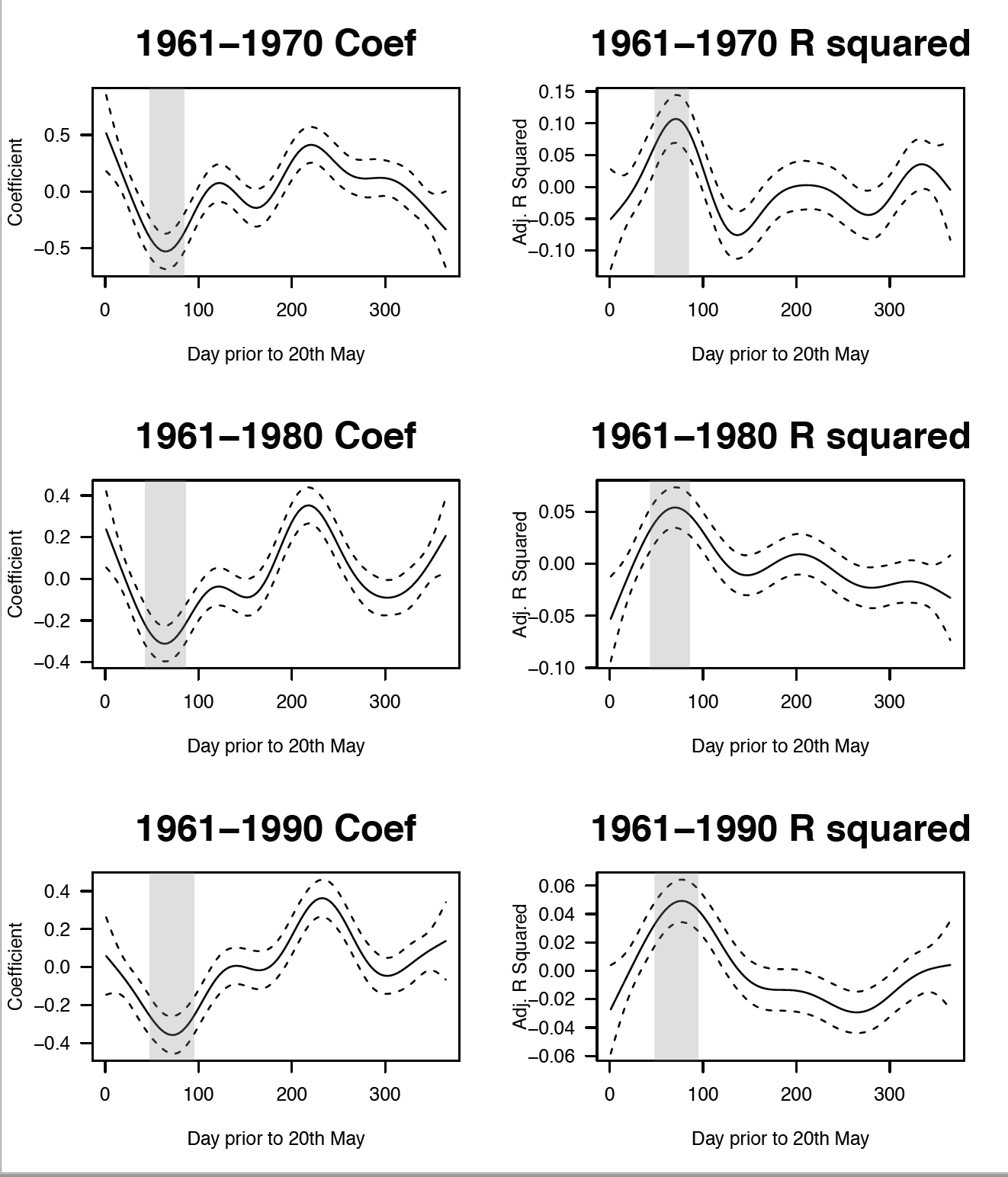


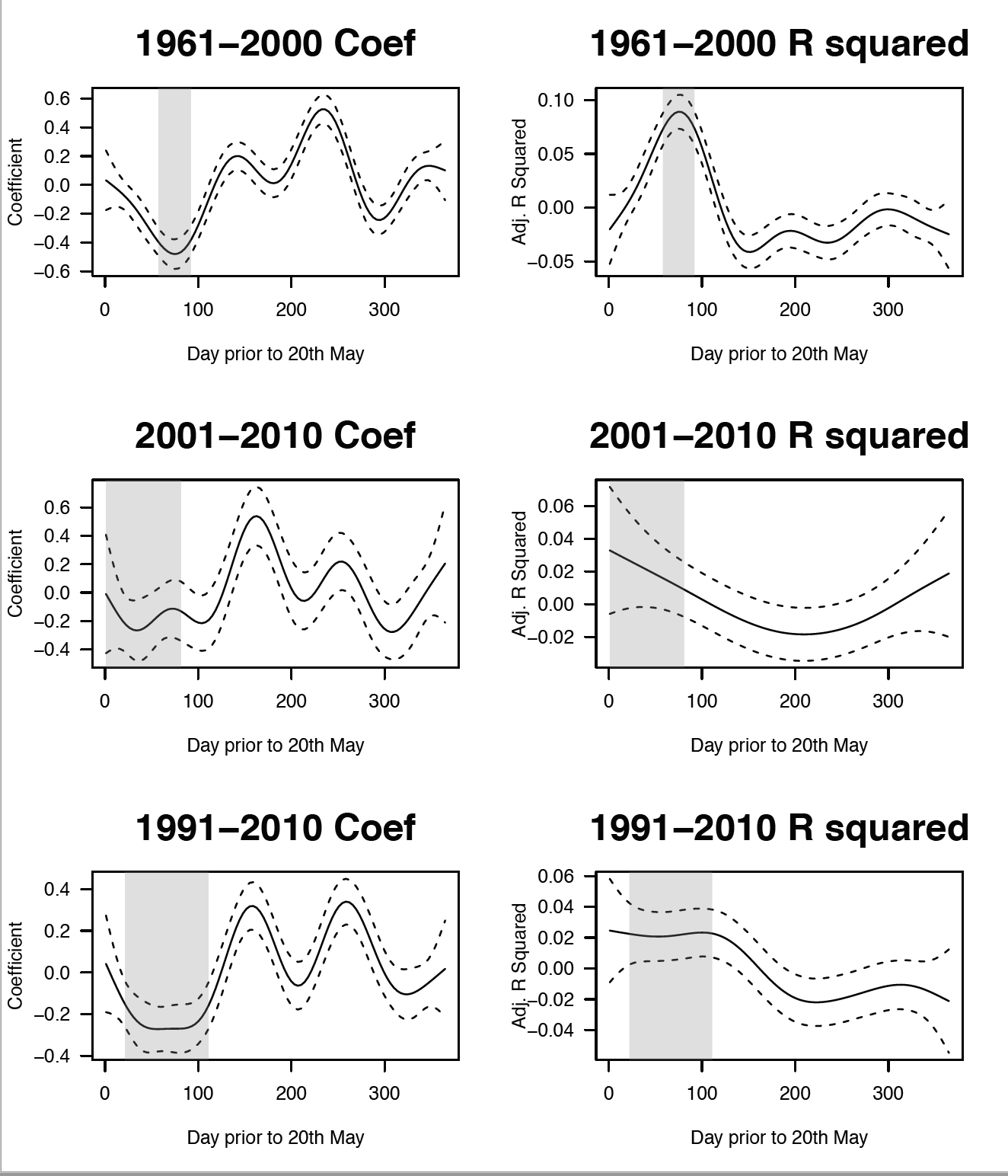


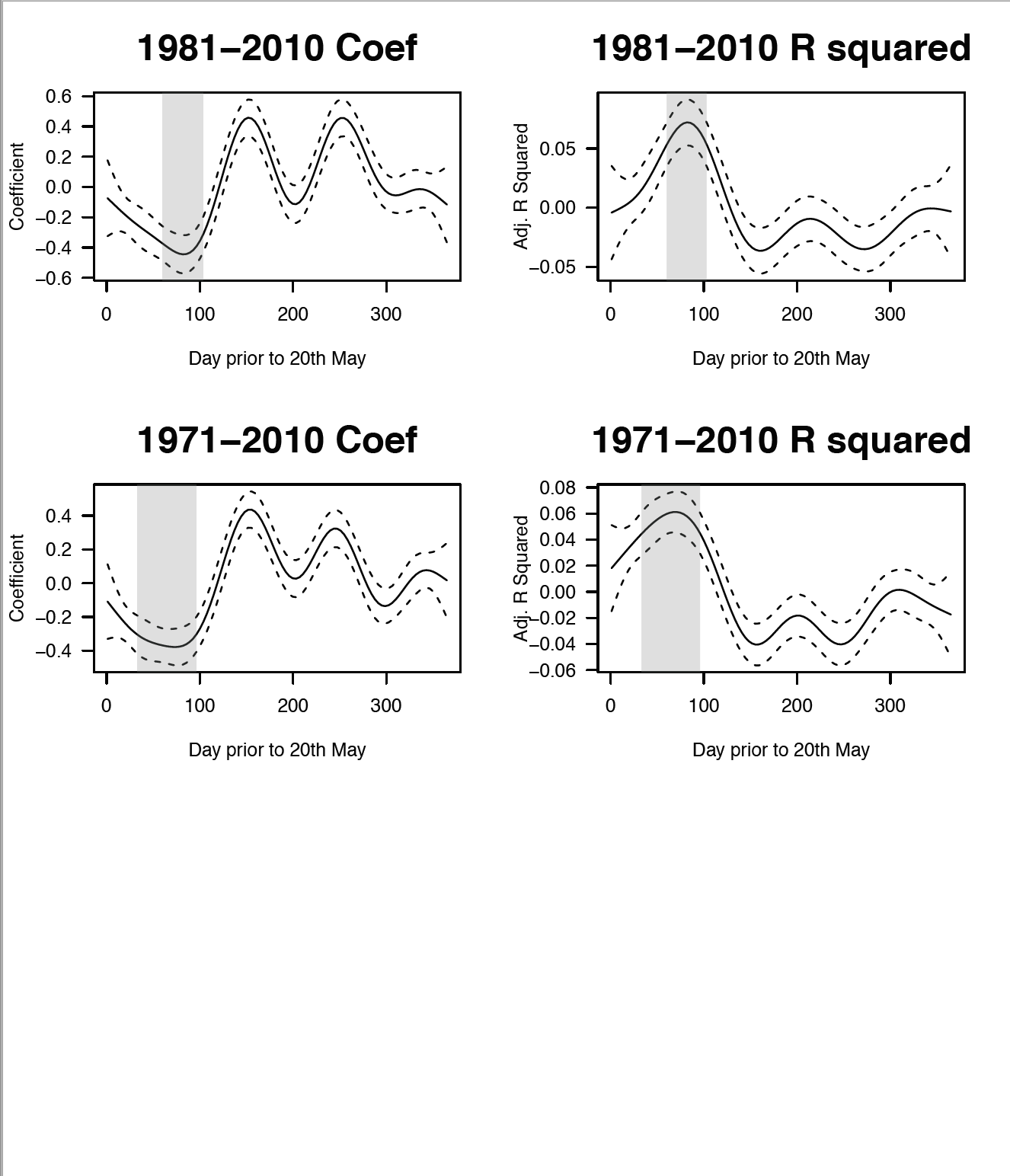


Figure S1: The figures below show the smoothed results of GAMs run on slope coefficient values and R^2^ values for each data subset in this study with annual mean lay date as the response variable.

The critical window, identified as the consecutive days which include the most extreme (greater or equal to the lowest 2.5 % and highest 97.5 %) R^2^ and slope coefficient values, is highlighted in grey.

##### DOY95 as response variable

Below we present the results of the GAMs performed on the slope coefficient and R^2^ values for each data subset when day of year 95 is the response variable.
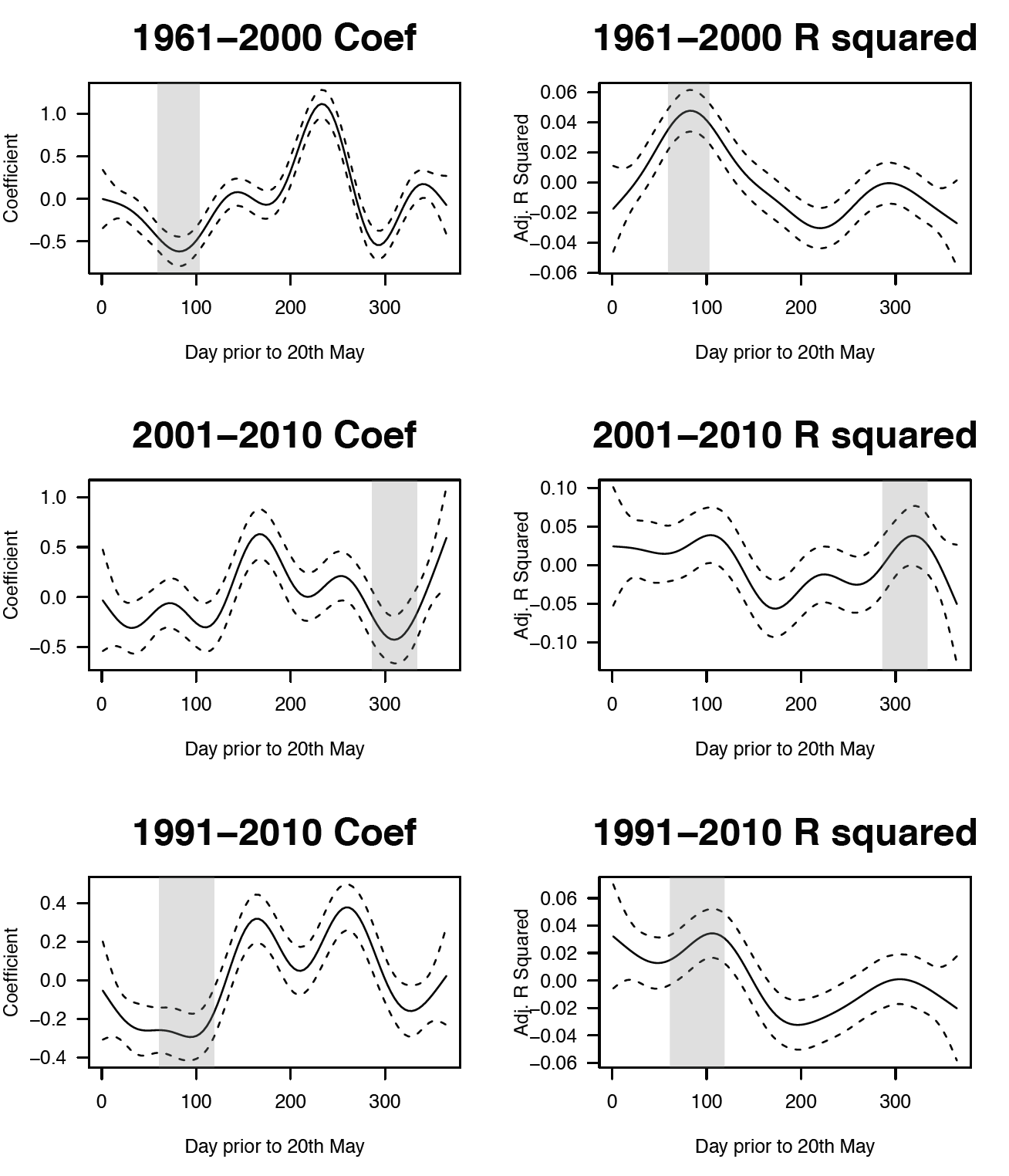


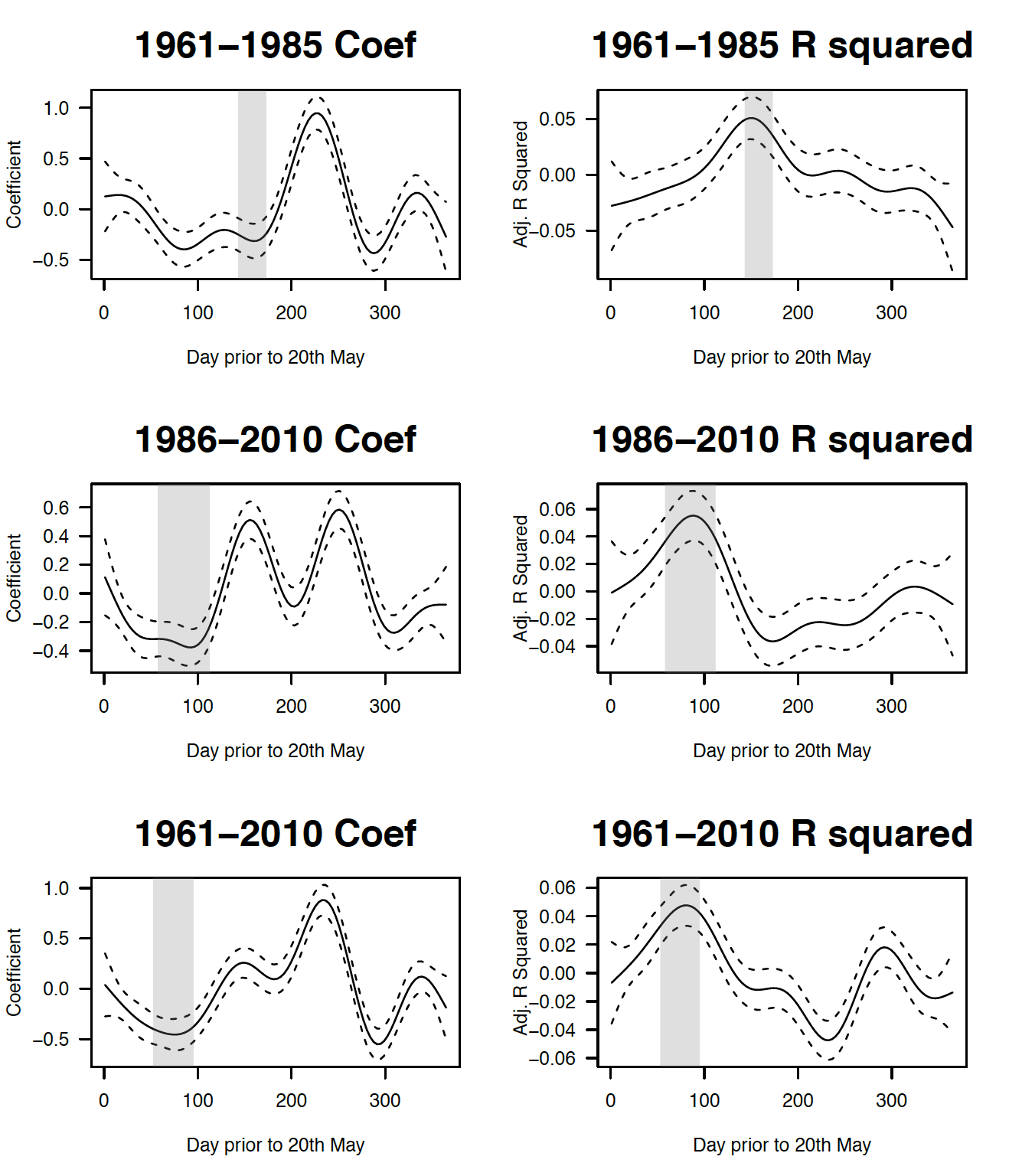


####
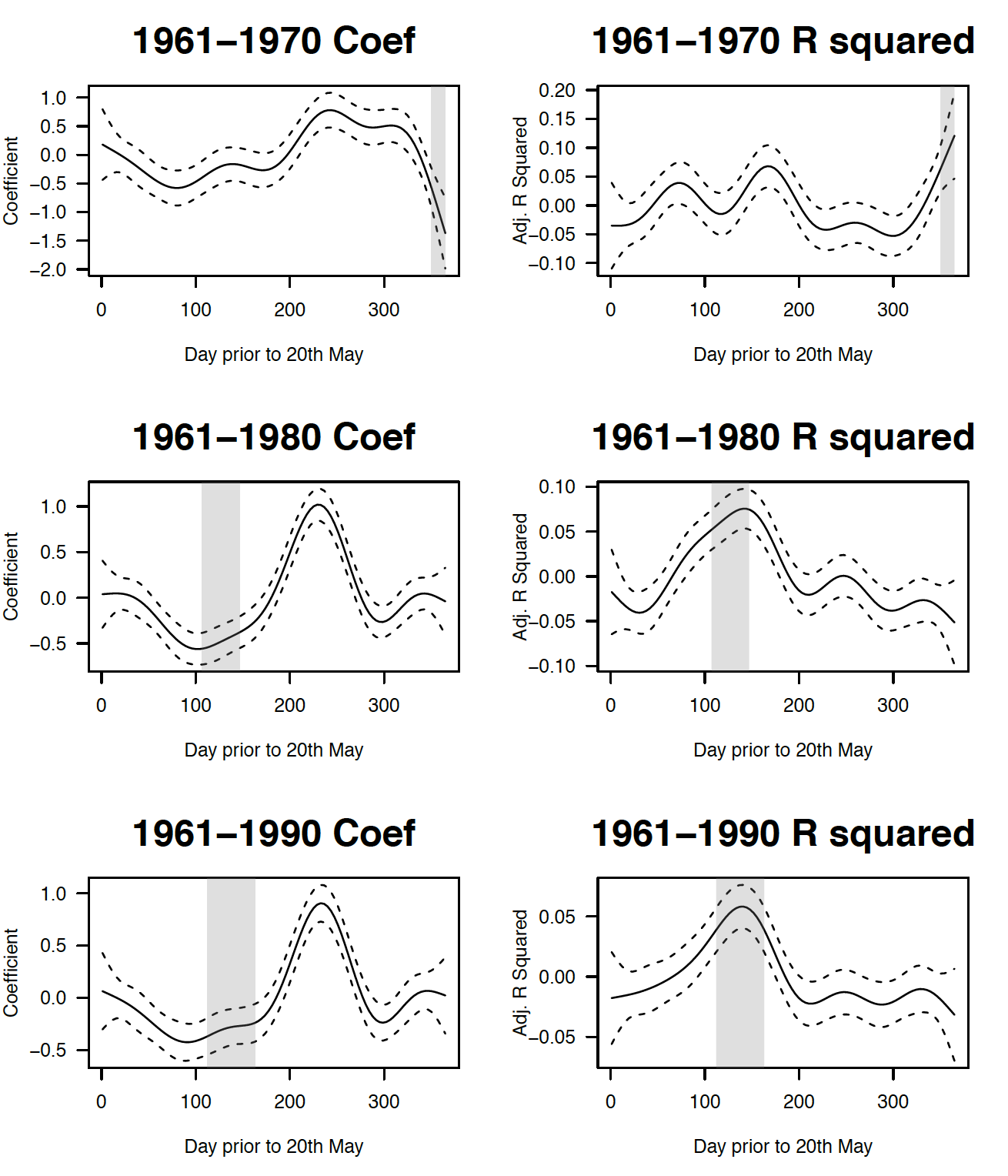


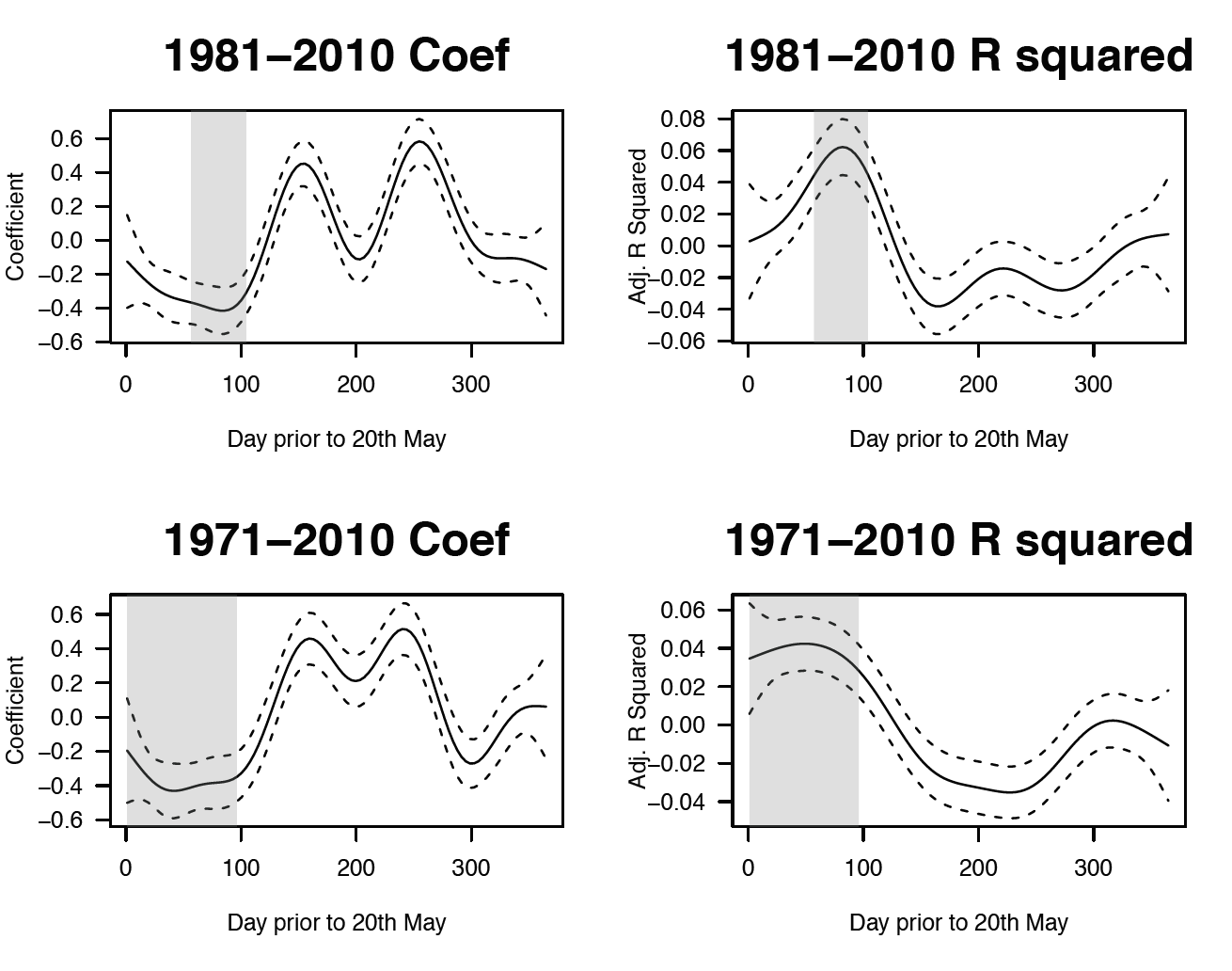


Figure S2: The figures below show the smoothed results of GAMs run on slope coefficient values and R^2^ values for each data subset in this study, with day of year 95 as the response variable.

The critical window, identified as the consecutive days which include the most extreme (greater or equal to the lowest 2.5 % and highest 97.5 %) R^2^ and slope coefficient values, is highlighted in grey.

#### S2 – Full GAM smooth results PSR

Below we present the full smoothed results of the P-spline GAM.
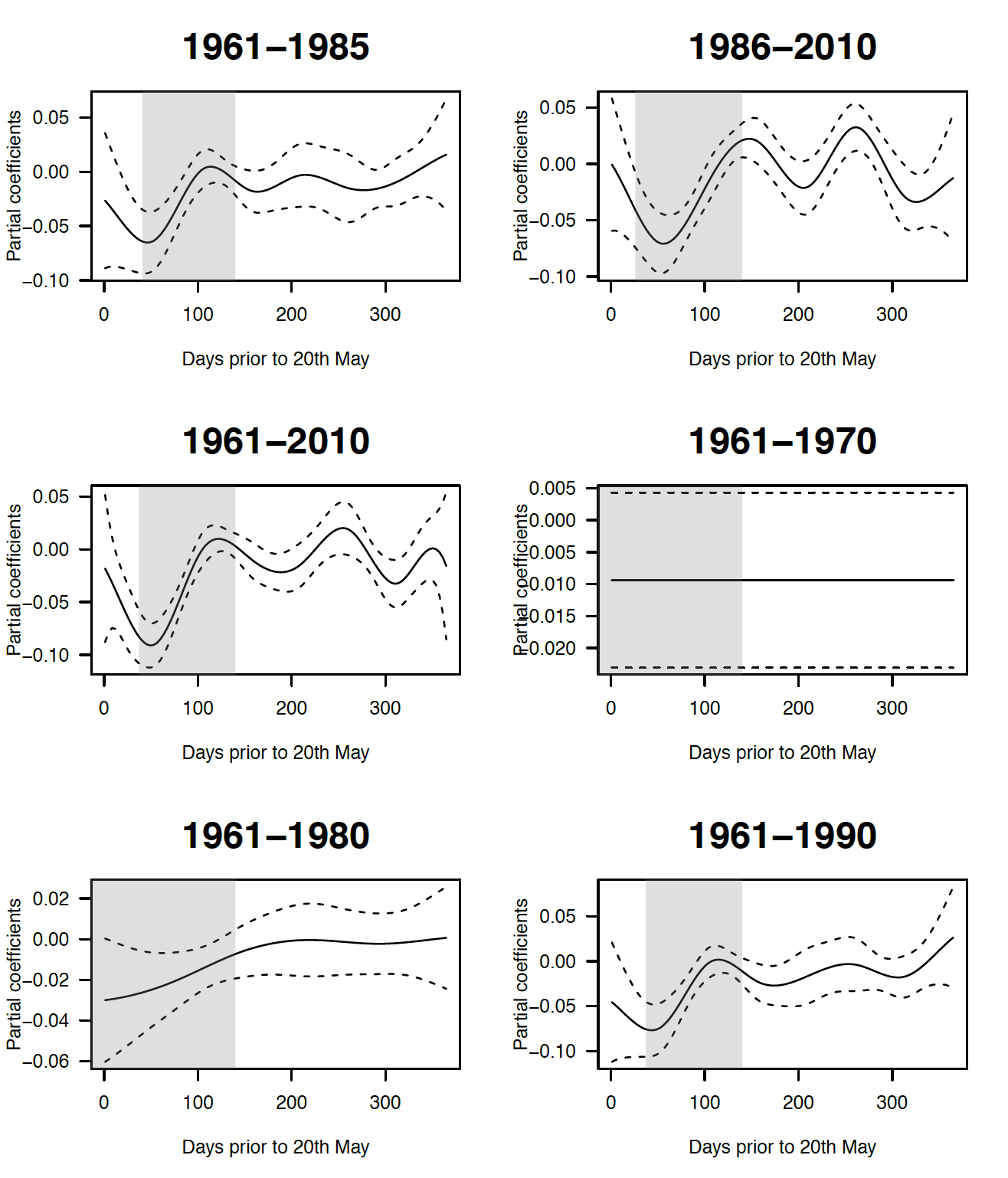


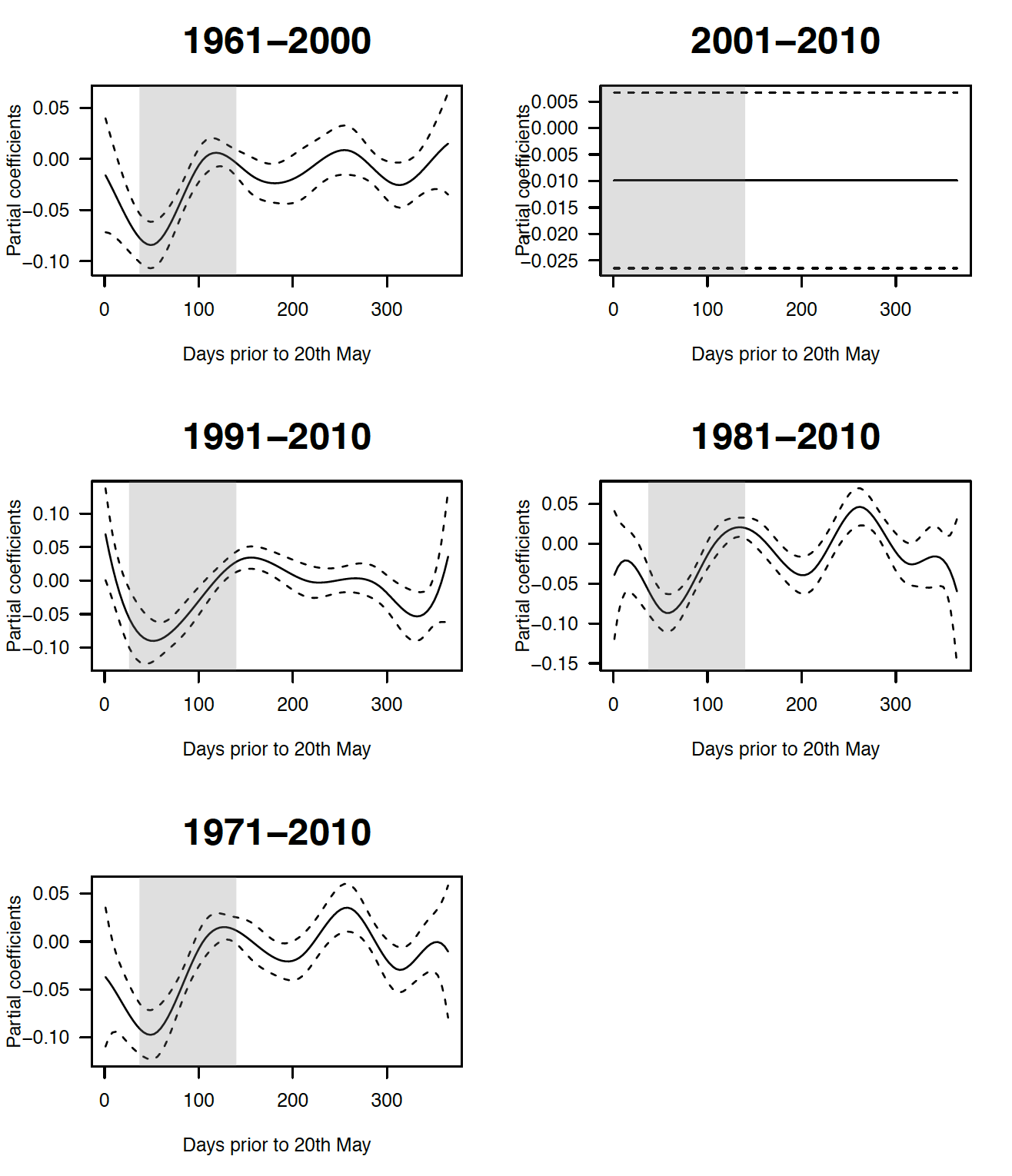


Figure S3: The figures below show the smoothed results of the P-spline GAM.

The critical window, identified as the consecutive days which include the most extreme (greater or equal to the lowest 2.5 % and highest 97.5 %) partial coefficient values, is highlighted in grey.

#### S3 – Plot of critical time windows using DOY95 as response variable for CSP

####
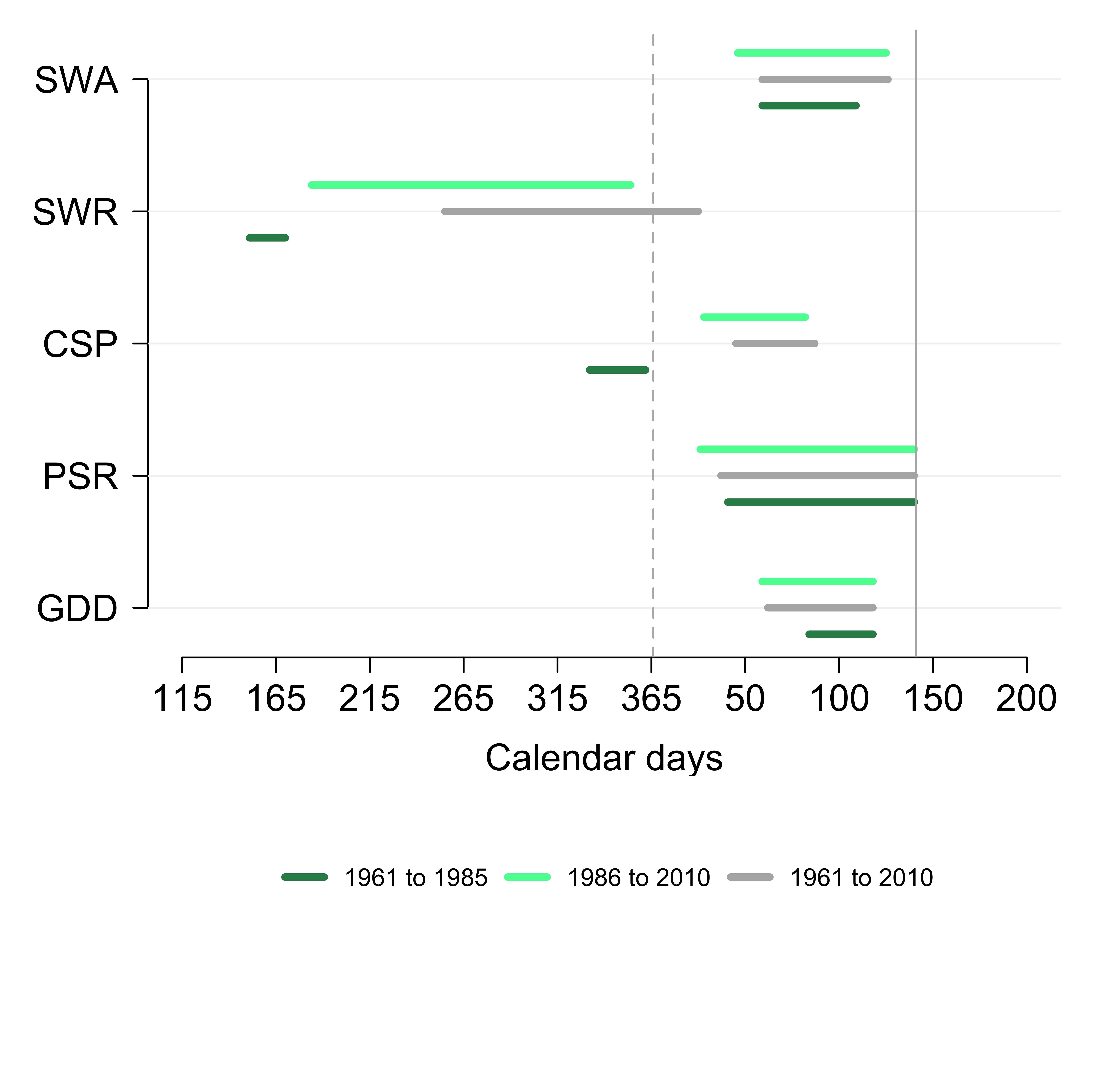


Figure S4: Temporal critical windows identified by different statistical methods and amounts of data.

Vertical dotted line shows the 1^st^ January and solid vertical line indicates 20^th^ May, the reference day for absolute methods (SWA, CSP, and PSR). GDD is plotted relative to the mean lay date across years and SWR relative to the reference date of 20^th^ May.

#### S4 – Full version of manuscript Table 1

Table S1: Summary statistics for linear models of identified cues and lay date for all methods.

Shows time period of data used, intercept of the regression, slope of the relationship, standard error (SE), adjusted R^2^ (R^2^), window open, and window close (in days prior to reference day). The minimum threshold temperature and cumulative GDD requirement are also presented.

| **Method** | **Time period** | **Aggregate statistic** | **Intercept** | **Slope** | **SE** | **R^2^** | **Window open** | **Window close** | |
| --- | --- | --- | --- | --- | --- | --- | --- | --- | --- |
| **SWA** | Early | mean | 140.66 | -2.83 | 0.54 | 0.53 | 81 | 31 | |
| **SWA** | Late | mean | 150.64 | -4.92 | 0.72 | 0.65 | 94 | 15 | |
| **SWA** | Whole dataset | mean | 163.91 | -6.06 | 0.54 | 0.72 | 81 | 14 | |
| **SWA** | 1961-1970 | mean | 152.59 | -4.11 | 0.45 | 0.90 | 72 | 25 | |
| **SWA** | 1961-1980 | mean | 150.77 | -3.65 | 0.65 | 0.61 | 58 | 24 | |
| **SWA** | 1961-1990 | mean | 160.77 | -5.38 | 0.85 | 0.57 | 81 | 11 | |
| **SWA** | 1961-2000 | mean | 161.47 | -5.67 | 0.63 | 0.67 | 81 | 14 | |
| **SWA** | 2001-2010 | slope | 112.74 | -49.28 | 6.98 | 0.84 | 339 | 313 | |
| **SWA** | 1991-2010 | slope | 112.81 | -5.72 | 0.89 | 0.68 | 8 | 2 | |
| **SWA** | 1981-2010 | mean | 157.51 | -5.59 | 0.68 | 0.69 | 87 | 15 | |
| **SWA** | 1971-2010 | mean | 164.88 | -6.27 | 0.60 | 0.74 | 81 | 14 | |
| **SWR** | Early | slope | 121.89 | 14.62 | 2.92 | 0.50 | 354 | 335 | |
| **SWR** | Late | slope | 87.25 | -443.71 | 66.90 | 0.64 | 321 | 151 | |
| **SWR** | Whole dataset | mean | 167.33 | -5.24 | 0.49 | 0.69 | 250 | 115 | |
| **SWR** | 1961-1970 | mean | 153.55 | -4.64 | 0.59 | 0.87 | 54 | 22 | |
| **SWR** | 1961-1980 | slope | 121.91 | 19.44 | 4.09 | 0.53 | 355 | 333 | |
| **SWR** | 1961-1990 | slope | 110.31 | -418.06 | 63.18 | 0.60 | 365 | 157 | |
| **SWR** | 1961-2000 | mean | 170.31 | -5.47 | 0.63 | 0.65 | 250 | 120 | |
| **SWR** | 2001-2010 | slope | 168.53 | -3.86 | 0.49 | 0.87 | 244 | 240 | |
| **SWR** | 1991-2010 | slope | 96.57 | -471.85 | 87.18 | 0.60 | 365 | 125 | |
| **SWR** | 1981-2010 | slope | 94.94 | -357.80 | 45.62 | 0.68 | 321 | 161 | |
| **SWR** | 1971-2010 | mean | 173.03 | -5.52 | 0.65 | 0.65 | 260 | 115 | |
| **CSP** | Early | mean | 131.63 | -1.46 | 0.51 | 0.23 | 73 | 48 | |
| **CSP** | Late | mean | 126.81 | -2.41 | 0.56 | 0.42 | 108 | 61 | |
| **CSP** | Whole dataset | mean | 135.78 | -3.06 | 0.52 | 0.41 | 90 | 51 | |
| **CSP** | 1961-1970 | mean | 133.62 | -1.83 | 0.67 | 0.42 | 85 | 48 | |
| **CSP** | 1961-1980 | mean | 132.18 | -1.57 | 0.59 | 0.24 | 86 | 43 | |
| **CSP** | 1961-1990 | mean | 133.73 | -2.34 | 0.57 | 0.35 | 95 | 48 | |
| **CSP** | 1961-2000 | mean | 132.21 | -2.34 | 0.47 | 0.38 | 92 | 58 | |
| **CSP** | 2001-2010 | mean | 173.21 | -6.88 | 2.05 | 0.53 | 81 | 1 | |
| **CSP** | 1991-2010 | mean | 141.98 | -4.25 | 0.98 | 0.48 | 111 | 22 | |
| **CSP** | 1981-2010 | mean | 127.94 | -2.47 | 0.52 | 0.43 | 103 | 60 | |
| **CSP** | 1971-2010 | mean | 145.18 | -4.56 | 0.57 | 0.62 | 96 | 33 | |
| **PSR** | Early | daily mean | 174.28 | NA | NA | 0.49 | 99.27 | 0.00 | |
| **PSR** | Late | daily mean | 159.24 | NA | NA | 0.82 | 113.98 | 0.00 | |
| **PSR** | Whole dataset | daily mean | 181.08 | NA | NA | 0.77 | 102.95 | 0.00 | |
| **PSR** | 1961-1970 | daily mean | 156.07 | NA | NA | 0.09 | 364.00 | 0.00 | |
| **PSR** | 1961-1980 | daily mean | 146.80 | NA | NA | 0.31 | 349.29 | 0.00 | |
| **PSR** | 1961-1990 | daily mean | 186.50 | NA | NA | 0.60 | 102.95 | 0.00 | |
| **PSR** | 1961-2000 | daily mean | 181.93 | NA | NA | 0.71 | 102.95 | 0.00 | |
| **PSR** | 2001-2010 | daily mean | 150.24 | NA | NA | 0.04 | 364.00 | 0.00 | |
| **PSR** | 1991-2010 | daily mean | 178.33 | NA | NA | 0.78 | 113.98 | 0.00 | |
| **PSR** | 1981-2010 | daily mean | 159.98 | NA | NA | 0.85 | 102.95 | 0.00 | |
| **PSR** | 1971-2010 | daily mean | 170.05 | NA | NA | 0.79 | 102.95 | 0.00 | |
|  |  |  |  |  |  |  |  |  | |
|  |  |  |  |  |  |  |  | **ºC min** | **ºC tot** |
| **GDD** | Early | sum | 10.06 | 0.66 | 0.09 | 0.91 | 84.06 | 1.43 | 247.37 |
| **GDD** | Late | sum | 0.91 | 10.06 | 0.66 | 0.09 | 58.86 | 1.30 | 341.39 |
| **GDD** | Whole dataset | sum | 0.09 | 0.91 | 10.06 | 0.66 | 61.53 | 1.03 | 355.50 |
| **GDD** | 1961-1970 | sum | 0.66 | 0.09 | 0.91 | 10.06 | 72.28 | 1.50 | 319.49 |
| **GDD** | 1961-1980 | sum | 10.06 | 0.66 | 0.09 | 0.91 | 85.80 | 2.43 | 203.37 |
| **GDD** | 1961-1990 | sum | 0.91 | 10.06 | 0.66 | 0.09 | 82.57 | 1.58 | 244.59 |
| **GDD** | 1961-2000 | sum | 0.09 | 0.91 | 10.06 | 0.66 | 56.51 | 1.00 | 376.79 |
| **GDD** | 2001-2010 | sum | 0.66 | 0.09 | 0.91 | 10.06 | 61.67 | 2.20 | 279.44 |
| **GDD** | 1991-2010 | sum | 10.06 | 0.66 | 0.09 | 0.91 | 68.98 | 2.30 | 257.30 |
| **GDD** | 1981-2010 | sum | 0.91 | 10.06 | 0.66 | 0.09 | 72.90 | 1.85 | 255.56 |
| **GDD** | 1971-2010 | sum | 0.09 | 0.91 | 10.06 | 0.66 | 71.77 | 1.81 | 267.16 |

#### S5 – Summary of predictive precision from all data subsets with different temporal distance between training data and prediction

Table S2: Summary of predictive precision of predictions from different dataset lengths and distance between training dataset and predictions

| **Method** | **Data length** | **Years between data and predicted years** | **PI width** | **% observations in PI** |
| --- | --- | --- | --- | --- |
| **SWA** | 50 | 0 | 15.15 | 100 |
| **SWA** | 40 | 0 | 8.18 | 0 |
| **SWA** | 30 | 0 | 11.98 | 40 |
| **SWA** | 20 | 0 | 15.93 | 80 |
| **SWA** | 10 | 0 | 15.40 | 80 |
| **SWA** | 40 | 10 | 10.95 | 80 |
| **SWA** | 30 | 20 | 12.23 | 80 |
| **SWA** | 20 | 30 | 14.88 | 100 |
| **SWA** | 10 | 40 | 14.38 | 100 |
| **SWA** | 40 | 0 | 7.03 | 20 |
| **SWA** | 30 | 0 | 10.42 | 20 |
| **SWA** | 20 | 0 | 15.15 | 40 |
| **SWA** | 10 | 0 | 15.14 | 20 |
| **CSP** | 50 | 0 | 21.82 | 100 |
| **CSP** | 40 | 0 | 18.17 | 20 |
| **CSP** | 30 | 0 | 15.57 | 20 |
| **CSP** | 20 | 0 | 18.88 | 60 |
| **CSP** | 10 | 0 | 20.64 | 80 |
| **CSP** | 40 | 10 | 21.20 | 100 |
| **CSP** | 30 | 20 | 16.16 | 100 |
| **CSP** | 20 | 30 | 19.73 | 100 |
| **CSP** | 10 | 40 | 17.32 | 100 |
| **CSP** | 40 | 0 | 16.76 | 20 |
| **CSP** | 30 | 0 | 14.53 | 20 |
| **CSP** | 20 | 0 | 18.40 | 20 |
| **CSP** | 10 | 0 | 20.46 | 100 |
| **PSR** | 50 | 0 | 7.81 | 60 |
| **PSR** | 40 | 0 | 12.82 | 20 |
| **PSR** | 30 | 0 | 9.86 | 20 |
| **PSR** | 20 | 0 | 12.03 | 80 |
| **PSR** | 10 | 0 | 8.88 | 80 |
| **PSR** | 40 | 10 | 9.31 | 40 |
| **PSR** | 30 | 20 | 9.42 | 40 |
| **PSR** | 20 | 30 | 7.92 | 60 |
| **PSR** | 10 | 40 | 8.03 | 60 |
| **PSR** | 40 | 0 | 6.90 | 20 |
| **PSR** | 30 | 0 | 5.93 | 20 |
| **PSR** | 20 | 0 | 9.05 | 0 |
| **PSR** | 10 | 0 | 6.44 | 0 |
| **GDD** | 50 | 0 | 67.60 | 100 |
| **GDD** | 40 | 0 | 62.60 | 100 |
| **GDD** | 30 | 0 | 72.20 | 100 |
| **GDD** | 20 | 0 | 63.60 | 100 |
| **GDD** | 10 | 0 | 61.80 | 100 |
| **GDD** | 40 | 10 | 117.40 | 100 |
| **GDD** | 30 | 20 | 80.00 | 100 |
| **GDD** | 20 | 30 | 82.00 | 100 |
| **GDD** | 10 | 40 | 75.40 | 100 |
| **GDD** | 40 | 0 | 67.00 | 80 |
| **GDD** | 30 | 0 | 66.20 | 80 |
| **GDD** | 20 | 0 | 83.40 | 80 |
| **GDD** | 10 | 0 | 92.20 | 100 |
